# App-SpaM: phylogenetic placement of short reads without sequence alignment

**DOI:** 10.1093/bioadv/vbab027

**Published:** 2021-10-13

**Authors:** Matthias Blanke, Burkhard Morgenstern

**Affiliations:** 1 Department of Bioinformatics, Institute of Microbiology and Genetics, Georg-August-University Göttingen, Göttingen 37077, Germany; 2 International Max Planck Research School for Genome Science, Göttingen 37077, Germany; 3 Campus-Institute Data Science (CIDAS), Göttingen 37077, Germany

## Abstract

**Motivation:**

Phylogenetic placement is the task of placing a query sequence of unknown taxonomic origin into a given phylogenetic tree of a set of reference sequences. A major field of application of such methods is, for example, the taxonomic identification of reads in metabarcoding or metagenomic studies. Several approaches to phylogenetic placement have been proposed in recent years. The most accurate of them requires a multiple sequence alignment of the references as input. However, calculating multiple alignments is not only time-consuming but also limits the applicability of these approaches.

**Results:**

Herein, we propose *Alignment-free phylogenetic placement algorithm based on Spaced-word Matches* (*App-SpaM*), an efficient algorithm for the phylogenetic placement of short sequencing reads on a tree of a set of reference sequences. *App-SpaM* produces results of high quality that are on a par with the best available approaches to phylogenetic placement, while our software is two orders of magnitude faster than these existing methods. Our approach neither requires a multiple alignment of the reference sequences nor alignments of the queries to the references. This enables *App-SpaM* to perform phylogenetic placement on a broad variety of datasets.

**Availability and implementation:**

The source code of *App-SpaM* is freely available on Github at https://github.com/matthiasblanke/App-SpaM together with detailed instructions for installation and settings. *App-SpaM* is furthermore available as a Conda-package on the Bioconda channel.

**Contact:**

matthias.blanke@biologie.uni-goettingen.de

**Supplementary information:**

Supplementary data are available at *Bioinformatics Advances* online.

## 1 Introduction 

Phylogeny reconstruction is a fundamental field of research in bioinformatics (Felsenstein, 2004). Here, the basic task is to reconstruct a phylogenetic tree for a set of nucleic acid or protein sequences, representing their evolutionary history. However, the *de novo* reconstruction of phylogenetic trees is resource-intensive and requires a well-curated set of biological sequences (Kapli *et al.*, 2020). But if a reliable phylogenetic tree is already known for a subset of the input sequences, then it is possible to efficiently find the position of the remaining sequences within this existing tree. This procedure is called *phylogenetic placement* and a number of algorithms have been proposed for this task during the last years (Barbera *et al.*, 2019; Brown and Truszkowski, 2013; Czech *et al.*, 2019; Matsen *et al.*, 2012; Mirarab *et al.*, 2012; Rabiee and Mirarab, 2020). By now, phylogenetic placement is a common step in metabarcoding studies for purposes such as taxonomic identification and microbiome analyses (Darling *et al.*, 2014; DeSalle and Goldstein, 2019; Mahé *et al.*, 2017; Nguyen *et al.*, 2014; Thompson *et al.*, 2017). This development is also facilitated by large curated databases for marker genes such as 16S (DeSantis *et al.*, 2006; Quast *et al.*, 2013) or ITS2 (Ankenbrand *et al.*, 2015) that make reference sequences readily available. It has also been argued that phylogenetic placement is more accurate than taxonomic read assignment (Barbera *et al.*, 2019) and a variety of tools has been developed to analyse and visualize resulting placements between different samples (Barbera *et al.*, 2021; Czech and Stamatakis, 2019; Czech *et al.*, 2020; Matsen and Evans, 2013). The phylogenetic placement has also been used to update large phylogenetic trees (Balaban *et al.*, 2021) and for the tracking of virus variants (Singer *et al.*, 2020; Turakhia *et al.*, 2021). 

The first approaches to phylogenetic placement were *pplacer* (Matsen *et al.*, 2010) and *EPA* (Berger *et al.*, 2011). Both programs are based on probabilistic models of nucleotide substitutions. For a set of reference sequences, they require a *multiple alignment* of these sequences—the *reference MSA*—together with the *reference tree*. For a query read and a position in the tree, *pplacer* calculates the *likelihood* for observing the read at this position. The program then finds a position that maximizes this likelihood. It uses several heuristics to rapidly calculate the likelihood values and has a runtime linear with respect to the number of reference taxa, number of queries and sequence lengths. Similar to *pplacer*, *EPA* also calculates the likelihood for each query read at each possible edge in the reference tree. *EPA-ng* (Barbera *et al.*, 2019) is a re-implementation of *EPA* designed to parallelize the computations and comes with additional improved placement heuristics. In addition to the reference alignment, all of the above approaches also require alignments of the query reads against the reference MSA. This alignment is often performed with *hmmalign* (Eddy, 1995; Finn *et al.*, 2011). Alternatively, phylogeny-aware alignment algorithms such as *PaPaRa* (Berger and Stamatakis, 2011) or *SEPP* (Mirarab *et al.*, 2012) can be used.

In comparison to *pplacer* and *EPA*, the more recently developed algorithm *RAPPAS* (Linard *et al.*, 2019) does not align the read sequences to the reference alignment. Instead, *RAPPAS* uses so-called *phylo-k-mers*, which are calculated based on the reference tree and reference MSA in a *pre-processing* step. For each column of the reference alignment, for each edge *e* of the reference tree, and for each possible *k*-mer *w*, the program calculates the probability to see *w* at the corresponding position in a hypothetical sequence that would branch off from the reference tree at edge *e* (note that *w* does not need to be present in any of the reference sequences). If this probability is above a chosen threshold, the *k*-mer *w* is called a phylo-*k*-mer. *RAPPAS* creates a database with all phylo-*k*-mers and their associated probabilities to occur at a branch in the reference tree. Once this database is constructed, new query reads can be rapidly placed based on their *k*-mers that are also present in the database.

In contrast to the above methods, the recently proposed algorithm *APPLES* (Balaban *et al.*, 2020) is a *distance-based* approach. The program chooses placement positions based on estimated phylogenetic distances between the reference sequences and the query read sequences. Then, *APPLES* finds a placement position in the reference tree such that the distances between the query and the reference sequences in the resulting tree approach the estimated phylogenetic distances. Here, a standard sum-of-squares criterion is applied. The distances between query and reference sequences can either be estimated from sequence alignments or by using an alignment-free method; in the latter case, *APPLES* uses *Skmer* (Sarmashghi *et al.*, 2019).

In this paper, we present a new approach to phylogenetic placement that we call *Alignment-free phylogenetic placement algorithm based on Spaced-word Matches* (*App-SpaM*). Similar to *APPLES*, *App-SpaM* also performs distance-based phylogenetic placement; it neither needs a multiple alignment of the reference sequences nor alignments of the query reads to the reference sequences. Thus, it skips the time intensive alignment procedures that are needed for most other software tools. Additionally, in contrast to existing placement methods, *App-SpaM* can be applied to datasets where no multiple sequence alignment of the references can be created.

*App-SpaM* is based on an approach that was originally implemented in the program *Filtered spaced-word Matches* (*FSWM*; Leimeister *et al.*, 2017): For any two of the input DNA sequences, *FSWM* estimates their Jukes–Cantor distances, i.e. the average number of nucleotide substitutions per position since the two sequences have evolved from their last common ancestor. This estimate is based on simple gap-free alignments of a fixed length, so-called *spaced-word matches*, that are created with respect to a pre-defined binary pattern of *match* and *don’tcare* positions. This concept has already been extended to calculate distances between an assembled genome from one species and a set of unassembled reads from a second genome, or between sets of unassembled reads from two genomes. This adaptation of *FSWM* is called *Read-SpaM* (Lau *et al.*, 2019).

*App-SpaM* is a fast implementation of the FSWM approach designed to perform phylogenetic placement: given a set of reference sequences, a reference tree for these sequences and a set of reads, *App-SpaM* uses spaced-word matches to estimate pairwise *phylogenetic distances* between every query and every reference sequence. It then uses one of several fast heuristics to perform phylogenetic placement of the queries. The heuristics are either based on the calculated query-reference distances or based on the *number* of identified spaced-word matches. We show that *App-SpaM* achieves a placement accuracy that is comparable to alignment-based approaches on a variety of datasets while it is faster than those existing methods.

## 2 Materials and methods

As input, our approach takes a set of *N reference sequences*, a rooted and edge-weighted phylogenetic tree *T*—the *reference tree*—with *N* leaves, where each leaf is labelled with one of the reference sequences, and a set of *query read* sequences. Our algorithm can be divided into three consecutive steps: (i) First, we find so-called *spaced-word matches* between every query and every reference sequence; (ii) then, we estimate the *phylogenetic distance* between every query and reference sequence based on a ‘filtered’ subset of the identified spaced-word matches; (iii) at last, we determine a placement position for each query sequence in the reference tree *T*.

### 2.1 Definitions

For a set Σ of *characters* called the *alphabet*, a *sequence over Σ* is an ordered list of elements of Σ. For a sequence *S*, its length is denoted by |S|, and for i∈{1,…,|S|}, the *i*-th element of *S* is denoted by S[i]. The set of sequences of length *n* over Σ is denoted by $\Sigma^{n}$. In the following, we are considering sequences over the set {0, 1}—so-called *patterns*—over the *nucleotide alphabet* A={A,C,G,T}, and over the *extended nucleotide alphabet* $A^{*}=A\cup \{*\}$. Here, ‘*’ is a symbol not contained in A, a so-called *wildcard character*.

A spaced word is defined with respect to a given binary pattern $P\in {\left\{0,1\right\}}^{\ell }$ of length ℓ. A position *j* in the pattern is called a *match* position if P[j]=1 and a *don’t care* position if P[j]=0. The number of match positions in a pattern *P* is called the *weight* of *P*. A *spaced word W* with respect to *P* is defined as a sequence of length |P| over $A^{*}$, with W[i]∈A if and only if *i* is a match position of *P*. We say that a spaced word *W* occurs in a sequence *S* over A at some position *i*—or that (*W*, *i*) is a spaced-word occurrence in *S*—if S[i+j−1]=W[j] for all match positions *j* of *P*.

**Fig. 1.**
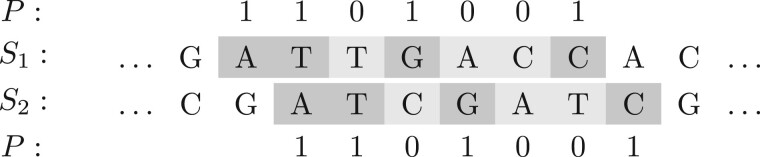
Toy example of a spaced-word match (SpaM) between two DNA sequences *S*_1_ and *S*_2_ with respect to a binary pattern *P *=* *1101001, representing *match positions* (‘1’) and *don’t care positions* (‘0’). The same spaced word AT*G**C occurs in both sequences. A *SpaM* corresponds to a local gap-free pairwise alignment with matching nucleotides at all *match positions* of *P*, while mismatches are allowed at the *don’t care* positions. Note that, in practice, we are using much larger patterns than in this example; by default, *App-SpaM* uses a single pattern with 12 match positions and 32 don’t care positions.

For two sequences *S*_1_ and *S*_2_ and positions *i*_1_ and *i*_2_ in *S*_1_ and *S*_2_, respectively, we say that there is a *spaced-word match* (*SpaM*) between *S*_1_ and *S*_2_ at $(i_{1},i_{2}$), if $S_{1}[i_{1}+j-1]=S_{2}[i_{2}+j-1]$ for all match positions *j* of *P*. In other words, there is a *SpaM* at (*i*_1_, *i*_2_), if there is a spaced word *W*, such that $\left(W,i_{1}\right)$ is a spaced-word occurrence in *S*_1_ and $\left(W,i_{2}\right)$ is a spaced-word occurrence in *S*_2_. A spaced-word match with respect to *P* can, thus, be seen as a local gap-free alignment of length |P| with matching nucleotides at the match positions of *P* and possible mismatches at the don’t care positions, see Figure  1 for an example. Furthermore, for a substitution matrix assigning a score to any two symbols of the nucleotide alphabet A, we define the *score* of a spaced-word match as the sum of all substitution scores of nucleotide pairs aligned to each other at the don’t care positions of *P*. Spaced-word matches—called *spaced seeds* in this context—have been originally introduced in sequence-database searching (Li *et al.*, 2003; Ma *et al.*, 2002); later they were applied in sequence classification (Břinda *et al.*, 2015) and alignment-free sequence comparison to estimate phylogenetic distances between DNA and protein sequences (Leimeister *et al.*, 2017, 2019; Morgenstern *et al.*, 2015; Röhling *et al.*, 2020), see Morgenstern (2020) for a review. The results of these methods depend on the underlying binary pattern or set of patterns. It is well known that finding optimal pattern sets is an *NP hard* problem (Li *et al.*, 2006), but efficient heuristics have been proposed for this task (Brejova *et al.*, 2004; Ilie *et al.*, 2011; Kucherov *et al.*, 2006).

### 2.2 Spaced-word matches between query and reference sequences

In a first step, we determine a fixed set P of binary patterns with a specified length and weight. For this, *App-SpaM* uses our previously developed software *rasbhari* to calculate pattern sets (Hahn *et al.*, 2016). By default, *App-SpaM* uses a single pattern, thus |P|=1, with a weight of *w *=* *12 and 32 don’t care positions. Given a pattern set P, first, we efficiently identify all spaced-word matches (*SpaMs*) between the query reads and the reference sequences. More precisely, for each pattern P∈P, all spaced-word occurrences with respect to *P* in the queries and references are stored in two lists *L*_1_ (references) and *L*_2_ (queries) that are both sorted in lexicographic order. For the sorting procedure, only the nucleotides at the *match* positions are considered, the *don’tcare* positions are ignored. For every spaced-word occurrence (*W*, *i*), we also store all nucleotides of the don’t care positions. Thus, for each possible spaced word *W*, all occurrences of *W* in the read and reference sequences appear as consecutive blocks in the lists *L*_1_ and *L*_2_, respectively.

Once the sorted lists *L*_1_ and *L*_2_ have been established, they can be traversed simultaneously, such that for each spaced word *W*, the blocks with the occurrences of *W* in *L*_1_ and *L*_2_ are processed at the same time. Each pair of occurrences $\left(W,i_{1}\right)$ in *L*_1_ and $\left(W,i_{2}\right)$ in *L*_2_ corresponds to a spaced-word match at (*i*_1_, *i*_2_) between a reference sequence and a query read. For each such spaced-word match, we calculate its score and we discard all *SpaMs* with a score smaller or equal to a pre-set threshold *t*. The *SpaMs* with a score below *t* are considered to be *background* or *random* spaced-word matches. All remaining, high-scoring, *SpaMs* are referred to as *filtered* spaced-word matches. These filtered spaced-word matches are regarded as putative *homologous* matches. As in *FSWM*, we use the *HOXD70* nucleotide substitution matrix (Chiaromonte *et al.*, 2002) and a default threshold value of *t *=* *0.

The above ‘filtering’ step is necessary since, in general, many of the spaced-word matches detected by our program will be random matches. In fact, for long input sequences with a low degree of similarity, the majority of the *SpaMs* will be background matches. Our inferred distance estimates are only unbiased if all background matches can be filtered out while all homologous matches remain. For dissimilar sequences, this assumption might not always hold and distances can be slightly biased depending on the threshold *t*; examples are given in our previous paper (Leimeister *et al.*, 2017). In this previous paper, however, we could also demonstrate that, with the filtering procedure, homologous and background SpaMs can be easily distinguished in most cases. The threshold *t *=* *0 that we are using by default works well for this purpose, but the value of *t* can be adapted by the user, if desired. There is a difference, however, between *App-SpaM* and the original program *FSWM*, in the way the filtered *SpaMs* are selected. In *FSWM*, each spaced-word occurrence $\left(W,i_{1}\right)$ in sequence *S*_1_ can be involved in at most one of the filtered spaced-word matches. In contrast, a spaced-word occurrence in a read sequence can be matched with multiple spaced-word occurrences in the reference sequences—and vice versa—in *App-SpaM*, as long as the corresponding scores are larger than *t*.

For each query read *Q* and each reference sequence *S*, we store the number *s*(*Q*, *S*) of *SpaMs* between *Q* and *S* with score larger than *t*. Additionally, we calculate the proportion of mismatches at the don’t care positions of all filtered *SpaMs* between *Q* and *S*, and we estimate the *phylogenetic distance d*(*Q*, *S*) between *Q* and *S* using the well-known *Jukes–Cantor* formula (Jukes and Cantor, 1969).

In practice, we compute the list *L*_1_ of spaced-word occurrences from the reference sequences once and hold it in main memory. The query sequences are processed in batches to limit the memory consumption. Thus, the list *L*_2_ of spaced-word occurrences in the query sequences is calculated and processed for each batch separately. This also allows straightforward parallelization for multi-core systems: multiple batches of query reads can be processed simultaneously across multiple cores for datasets with many query sequences.

### 2.3 Choosing a position for a read in the reference tree

In the following, we propose five heuristics to find a suitable position in our reference tree *T*, where a query read sequence *Q* is added to *T*. For an edge *e* in an edge-weighted tree, let *l*(*e*) denote the length (‘weight’) of *e*. For each query *Q*, we first select an edge *e_Q_* in *T* and insert a new internal node into this edge, thereby splitting *e_Q_* into two new edges *e*_1_ and *e*_2_ with $l(e_{1})+l(e_{2})=l(e_{Q})$. Then, we add a new leaf that is labelled with *Q*, together with a new edge $e_{Q}^{\prime }$ that connects this new leaf with the newly generated internal node. Finally, a length $l(e_{Q}^{\prime })$ is assigned to the newly generated edge $e_{Q}^{\prime }$.

To find a suitable edge *e_Q_* for a query sequence *Q* and to assign lengths to the newly generated edges, we are using either the phylogenetic distances *d*(*Q*, *S*) or the number of spaced-word matches *s*(*Q*, *S*) with scores larger than *t* between *Q* and all reference sequences *S*. A detailed description how we determine the edge lengths for *e*_1_, *e*_2_, and the newly inserted edge $e\prime_{Q}$ are given in the Supplementary Section S1.1. In case we find no spaced-word matches for a query read to any reference sequence, the query placement is recorded at the root of *T*.

#### 2.3.1 MIN-DIST

In this approach, we first select the reference sequence *S* that minimizes the distance *d*(*Q*, *S*) over all reference sequences, and we define *e_Q_* to be the edge in *T* that is adjacent to the leaf labelled with *S*. If multiple references have the same smallest distance to *Q*, one of them is chosen randomly.

#### 2.3.2 SpaM-COUNT

This works like the previous approach, but instead of selecting the reference sequence *S* that minimizes the distance to *Q*, we select the reference *S* that maximizes the number *s*(*Q*, *S*) of spaced-word matches with score >t between *S* and *Q*.

#### 2.3.3 LCA-DIST

Here, we identify the *two* reference sequences *S*_1_ and *S*_2_ with the lowest distances $d(Q,S_{1})$ and $d(Q,S_{2})$ to *Q*. Let *v* be the *lowest common ancestor* in *T* of the two leaves that are labelled with *S*_1_ and *S*_2_, respectively. The edge *e_Q_* is then defined as the edge in *T* that connects *v* with its parental node.

#### 2.3.4 LCA-COUNT

This is similar to the previous approach, but instead of using reference sequences *S*_1_ and *S*_2_ minimizing the distance with *Q*, we select the two references *S*_1_ and *S*_2_ with the maximal number $s(Q,S_{1})$ and $s(Q,S_{2})$ of spaced-word matches to *Q* with scores larger than *t*.

#### 2.3.5 SpaM-X

The first four approaches will yield either only placement locations at branches directly above the leaves, or only placement locations at inner branches, respectively. Thus, in this approach, we combine the *SpaM-COUNT* and *LCA-COUNT* approaches: For *S*_1_ and *S*_2_ as in *LCA-COUNT* and a given *X*, we evaluate whether

$$|s(Q,S_{1})-s(Q,S_{2})|>\frac{s(Q,S_{1})+s(Q,S_{2})}{X}$$

is true. If so, the query is placed according to *SpaM-COUNT*, otherwise, the query is placed according to *LCA-COUNT*. As a result, when $d(Q,S_{1})$ is substantially larger than $d(Q,S_{2})$, then *Q* is placed at the branch directly above *S*_1_. *App-SpaM* uses SpaM-X on default with *X *=* *4 (referred to as SpaM-4 in the following).

*SpaM+APPLES*: As a sixth approach, in addition to five versions of *App-SpaM*, we used the distance values *d*(*Q*, *S*) as input for the program *APPLES* (Balaban *et al.*, 2020). *APPLES* performs a least-squares optimization to find the position in the tree that fits the calculated distances best. For a reference tree *T*, a query sequence *Q* and input distances between *Q* and all reference sequences, it finds a position for *Q* in *T*, such that the sum of the squared differences between the input distances and the distances in the resulting tree is minimized.

### 2.4 Evaluation procedure

We primarily used the recently developed *Placement Evaluation WOrkflows (PEWO)* (Linard *et al.*, 2021) to evaluate the placement accuracy and runtime of *App-SpaM*. For a given reference dataset, consisting of a set of reference sequences, a reference MSA of these sequences, and a reference tree in which the leaves are labelled with the reference sequences, the *pruning-based accuracy evaluation* (*PAC*) implemented in *PEWO* determines the placement accuracy of an evaluated method as follows: First, a randomly chosen subtree is removed (‘pruned’) from the reference tree. All sequences at the leaves of the chosen subtree are removed from the reference MSA as well. Next, artificial reads are generated by splitting the removed sequences into segments of a given length; these reads are used as query sequences. An algorithm under evaluation is then used to place the queries onto the pruned reference tree.

To measure the placement accuracy of a method, *PEWO* uses the so-called *node distance* (*ND*): For each query sequence *Q*, the distance between the proposed placement position of *Q* and the edge where the subtree was pruned is measured by counting the number of nodes on the corresponding path. The *average* of these distances over all query sequences is then a measure of accuracy for one pruning event. This procedure is repeated with randomly pruned subtrees and *PEWO* uses the *average* accuracy over all pruning events as the overall accuracy of the evaluated method. *PEWO* also provides a *resources evaluation* (*RES*) workflow to measure the runtime and memory usage of programs. This includes the alignment of queries against the MSA of references for those methods that are based on sequence alignments and the construction of the phylo-*k*-mer database in *RAPPAS*.

We used the *PEWO PAC* workflow to evaluate the placement accuracy of the five versions of *App-SpaM* that we outlined above. In addition, we evaluated the combination of *SpaM* and *APPLES*. Both programs were run with default parameter values. We also ran our program with non-default parameter values for the pattern weight *w* and the number of patterns by varying w∈{8,12,16} and using between 1 and 5 different patterns per pattern set.

We compared the accuracy of *App-SpaM* to all programs that are currently supported by *PEWO* with the *PAC* workflow. At present, the programs *pplacer*, *EPA*, *EPA-ng*, *RAPPAS* and *APPLES* are included in the *PEWO* package. The datasets used for this evaluation vary with respect to the number and length of the reference sequences, with respect to the degree of similarity between the reference sequences, and with respect to the sequence locus. A short overview of the reference datasets is given in Table  1; a more detailed overview can be found in the Supplementary Section S1.3. In this analysis, in addition to the default parameters, we included a variety of parameter combinations for all placement programs. For *APPLES*, we used the updated version 2.0.1 that automatically re-estimates the branch lengths of the reference tree appropriately to match the evolutionary model of query-reference distances. For each reference dataset, we performed 100 pruning runs with randomly sampled subtrees and recorded the average *ND*. As default, we used a length of 150 base pairs (bp) for the simulated query reads. We also performed additional test runs with longer read lengths for three datasets (*hiv-104*, *neotrop-512* and *tara-3748*).

**Table 1.** Datasets used for evaluation

Name	Locus	Mean length (bp)	Query length (bp)
bac-150	16S	1256	150
hiv-104	Viral genomes	9096	150; 500
neotrop-512	16S	1766	150; 300
tara-3748	16S	1406	150; 300
bv-797	16S	1341	150
epa-218	16S	1483	150
epa-628	5.8S	780	150
epa-714	16S	1169	150
wol-43	Microbial genomes	52 768 066	150

CPU-652	16S	1315	150
CPU-512	16S	1766	150

*Notes*: Overview of datasets used in the evaluation. The columns show the dataset names used in this manuscript, the locus from which the sequences in the datasets originate from, the mean sequence length of the references and the simulated read lengths used during evaluation. The name of each dataset includes the number of reference sequences. The first nine datasets are used in the *PAC* workflow, the last two in the *RES* workflow.

To measure the runtime and memory requirements of the evaluated methods, we used *PEWO’s RES* workflow on two datasets of differing sizes (*CPU-652* and *CPU-512*). We recorded the average runtime and memory usage over five repeats for each program. For *CPU-652*, we placed 100 000 query sequences, and for *CPU-512*, we placed 10 000 query sequences on the reference tree. In addition to these evaluations, we performed an additional runtime test with *App-SpaM* on the *tara-3748* dataset, similarly to the runtime study conducted in *EPA-ng*: We recorded the runtime of *App-SpaM* to place up to 37 480 000 query reads with parallel execution on 30 cores. All of these tests were carried out on *Intel(R) Xeon(R) E7-4850* CPUs with 2 GHz.

Lastly, we also performed test runs using simulated sets of unassembled sequencing reads as references, instead of contiguous reference sequences. In these experiments, we used the *hiv-104* dataset that consists of complete *HIV* genomes, and *wol-43*, a dataset of complete microbial genomes of 43 different *Wolbachia* strains. Because *PEWO* does not support unassembled reference sequences, we used a simple leave-one-out procedure to assess the accuracy of *App-SpaM* in this scenario. For this, we simulated reads with a length of 150 bp and values for the sequencing coverage of 4, 2, 1, 0.5, 0.25, 0.125, 0.0625 and 0.03125 for all sequences. These ‘bags of reads’ constitute the reference sequences for the evaluation. Then, for a given coverage, a leaf is pruned from the reference tree, its corresponding reads are used as the query sequences, and the average *ND* across all reads is measured. This is repeated one by one for all references, and subsequently for all coverages. To assess the accuracy of *App-SpaM* in these tests, we used three control methods: First, we compared the placement accuracy to the accuracy achieved on contiguous (‘assembled’) reference sequences; this shows the decline in accuracy depending on the sequencing coverage. Second, we compared the achieved results to the accuracy when placing all query reads at the root of the tree; and third, when placing all query reads at the midpoint of the tree.

## 3 Results

**Fig. 2.**
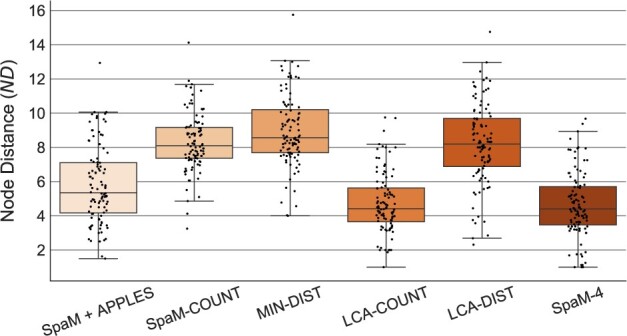
Average *ND* over *n *=* *100 pruning experiments on the *bac-150* dataset. *ND* was measured with *PEWO* for the five different versions of *App-SpaM* and the combination of *SpaM* and *APPLES*. A single pattern with weight *w *=* *12 and 32 don’t care positions is used for all heuristics. Every box plot shows the overall distribution over all 100 pruning experiments (black dots).

Figure  2 shows the accuracy of the five different versions of *App-SpaM* described above, together with the combination of *SpaM+APPLES*, on the *bac-150* dataset and a query read length of 150 bp. On average, *SpaM-4* performed best among the five methods while *MIN-DIST* was the least accurate. These results are consistent across different values of *w* and across different datasets (see Supplementary Section S2.1). In general, *w* has little influence on the placement accuracy of *App-SpaM* and there is no value of *w* that performs best in all situations. The second best of the five placement methods was *LCA-COUNT* followed by *SpaM+APPLES*, while *LCA-DIST*, *MIN-DIST* and *SpaM-COUNT* perform not as well.

**Fig. 3.**
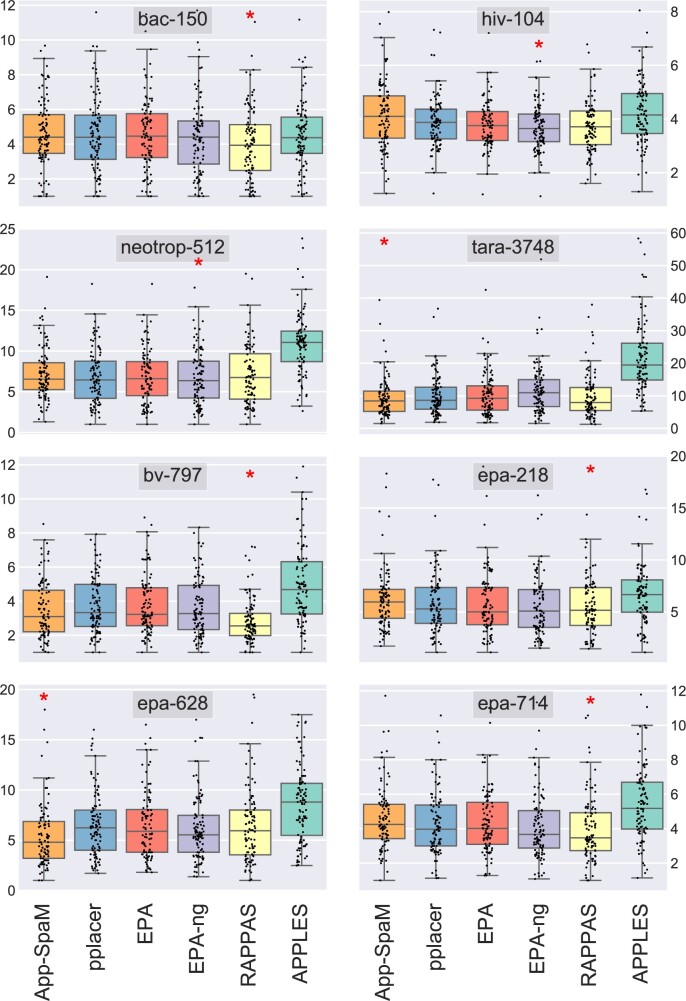
Average *ND* over *n *=* *100 pruning experiments on all eight datasets (grey patches) for all six programs (coloured box plots). The *y*-axes show the *ND*, the *x*-axes are divided into six categories that correspond to the six programs. Every box plot shows the overall distribution over all 100 pruning experiments (black dots). The program that performs best on average is highlighted (red star).

Figure  3 shows the *ND* for *App-SpaM* and five other placement methods on eight different datasets, again for a query read length of 150 bp. For each program, the placement accuracy for their default parameter settings is shown. *App-SpaM* is the most accurate program on the *tara-3748* and *epa-628* datasets, while *EPA-ng* performs best on the *hiv-104* and *neotrop-512* datasets; on the remaining four datasets, *RAPPAS* achieves the lowest average *ND* value among all evaluated programs. The exact statistics as well as results for other parameter settings for all programs are given in the Supplementary Material.

**Fig. 4.**
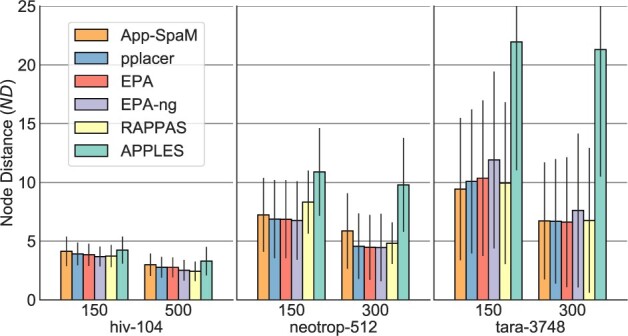
Average *ND* (*n *=* *100) for different placement programs, for different read lengths on three datasets. For *hiv-104* read lengths of 150 and 500 bp, and for *neotrop-512* and *tara-3748* results for read lengths of 150 and 300 bp were used. SDs across prunings are shown (black lines).

Figure  4 shows the performance of the evaluated methods for different query read lengths. Additionally, to read lengths of 150 bp, here, we used the dataset *hiv-104* with query read lengths of 500, and the datasets *neotrop-512* and *tara-3748* with query read lengths of 300. As expected, all programs are more accurate when longer query reads are used. The *ND* improves on average by 29% for *hiv-104*, by 27% for *neotrop-512* and by 24% for *tara-3748* across all methods, compared to a read length of 150 bp. In general, *likelihood*-based programs and RAPPAS benefit more from longer reads than *App-SpaM* and *APPLES*. For *likelihood*-based programs and RAPPAS, the *ND* drops on average by 30% across the three datasets when using longer query reads. In contrast, *App-SpaM* has a 25% lower *ND* and *APPLES* 12% lower *ND* on average across the three datasets when using longer queries.

**Fig. 5.**
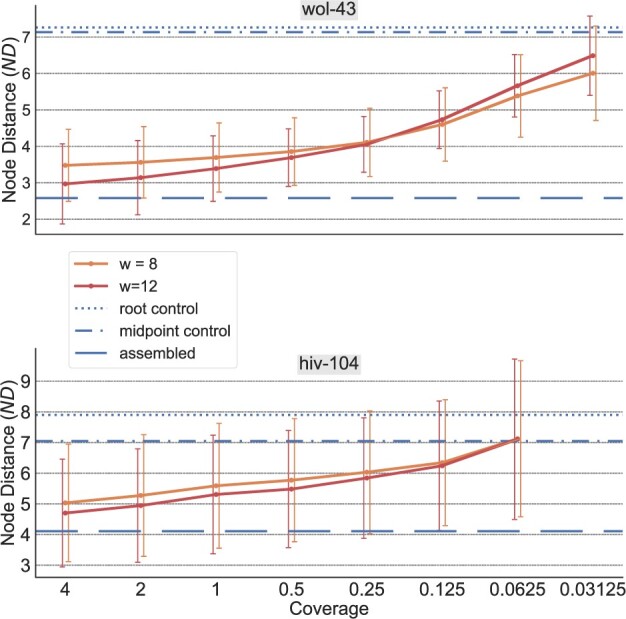
Average *ND* on unassembled reference sequences with different sequencing coverage on the *wol-43* (*n *=* *43) and *hiv-104* (*n *=* *104) datasets for *App-SpaM* with default parameters. Results for read lengths of 150 bp and a pattern *weight* of 8 and 12 are shown (orange and red) with SDs. Three control methods serve as reference for the performance of *App-SpaM*: The placement accuracy with assembled references (long dashed blue), always placing a query at the midpoint of the tree (dashed-dotted blue) and always placing a query at the root (dotted blue).

The accuracy of *App-SpaM* on unassembled reference sequences is shown in Figure  5 for different values of the sequencing coverage and for different values of the pattern weight *w*. For a coverage of 1, the *ND* for *App-SpaM* with *w *=* *12 increases—in comparison to assembled references—by 30% on *hiv-104* and by 31% on *wol-43*, respectively. The accuracy decreases for lower and increases for higher coverages of the reference sequences: With a coverage of 4, the ND is only 14% larger for *hiv-104* and 15% larger for *wol-43* in comparison to assembled references. On the *hiv-104* dataset and a coverage of 0.0625, *App-SpaM*’s inferred positions are only as good as the control method that always places a query read at the midpoint of the tree. However, with this low coverage of the viral genomes, every reference genome is, on average, only represented by 3.8 reads with a length of 150 bp. For the same reason, no results could be produced for a coverage of 0.03125 for *hiv-104*.

Runtime results for *CPU-652* and *CPU-512* are shown in Table  2. We report the runtimes for all pre-processing steps, the placement itself, as well as the total runtime (pre-processing plus placement). For some programs, the runtimes vary greatly depending on the chosen parameters. For *RAPPAS*, the pre-processing step—the assembly of the phylo-*k*-mer database—is relatively time-consuming. This database, however, needs to be assembled only once for a set of reference sequences. This is a considerable advantage of *RAPPAS* if multiple sets of query reads are placed on the same set of reference sequences. On these two datasets, *App-SpaM* performs 30–60 times faster than the next fastest program (*EPA-ng*) and 3–5 times faster than the placement step of *RAPPAS*. On *CPU-652*, *App-SpaM* has the lowest memory footprint and on *CPU-512* the second lowest behind *APPLES* (see Supplementary Section S3.4). In the large runtime example on the *tara-3748* dataset, *App-SpaM* placed a total of 37 480 000 queries in 613 min with parallel execution on 30 threads. When the pattern weight is increased to *w *=* *16 the runtime drops to 475 min.

**Table 2.** Runtime comparison of programs

	App-SpaM		RAPPAS		APPLES	EPA-ng	EPA	pplacer
	*w *=* *12	*w *=* *16	*k *=* *6	*k *=* *8				
CPU-652								
Pre-proc.	—	—	651	7253	3437	3437	3437	3437
Placement	152	79	710	454	2804	1315	194338	9257
Total	152	79	1361	7707	6241	4752	197775	12694
CPU-512								
Pre-proc.	—	—	1070	12144	1879	1879	1879	1879
Placement	34	22	254	185	348	127	6626	1976
Total	34	22	1324	12329	2227	2006	8505	3855

*Notes*: Comparison of runtimes for all tested programs on two datasets. 10 000 queries were placed for *CPU-512* and 100 000 queries for *CPU-652*. All runtimes are shown in seconds. For each dataset, we show the time for pre-processing (*pre-proc.*), *placement* and the *total* sum of pre-processing and placement with default parameters. Pre-processing includes generating the query alignment or building the phylo-*k*-mer database for *RAPPAS*.

## 4 Discussion

In this paper, we proposed a new method for phylogenetic placement called *App-SpaM*. To estimate phylogenetic distances, *App-SpaM* uses inexact word matches, so-called *SpaMs*, that are based on a binary pattern of *match* and *don’tcare* positions (Leimeister *et al.*, 2017). As previously shown, such spaced-word matches can be used to accurately estimate phylogenetic distances based on the number of mismatches per position at the *don’tcare* positions. In the present paper, we applied this approach to the problem of phylogenetic placement, by estimating distances between query and reference sequences.

We are using sets of *filtered SpaMs* as a substitute for full pairwise sequence alignments of the input sequences. A *SpaM* can be seen as a pairwise local alignment of a given length that, by definition, does not include gaps. Thus, strictly spoken, this approach can only deal with un-gapped homologies; insertions and deletions (indels) can introduce a certain bias in our approach: If a local homology contains an indel, it is possible that an *SpaM* correctly aligns homologous sequence positions to each other on one side of the indel, but then aligns non-homologous positions on the other side. Since the score of such an *SpaM* may still be above the filtering threshold, such ‘partial homologies’ can pass our filtering procedure and would then be used to estimate the phylogenetic distance between the two sequences. As a result, the average number of substitutions per position—i.e. the Jukes–Cantor distance of the sequences—would be over-estimated. We investigated the influence of indels on the accuracy of the estimated distances based on simulated sequence data (Leimeister *et al.*, 2017). The result was that, even in the presence of indels, our approach still provides fairly accurate distance estimates.

Our extensive evaluation shows that *App-SpaM*’s accuracy is close to the accuracy of the best-performing *likelihood*-based methods on most benchmark datasets that we used, see Figure  3. One exception is a set of complete viral genomes *(hiv-104)*, where *App-SpaM* performed not as accurate as the competing programs that we evaluated. In contrast, *App-SpaM* achieved the best placement accuracy out of all programs that we tested on two other datasets: a large set of 16S sequences (*tara-3748*) and a set of fungal 5.8S sequences (*epa-628*). The runtime of *App-SpaM* on these datasets was considerably faster than for the alternative methods.

We also presented five different placement heuristics for *App-SpaM* in this paper. Two of them performed best across all datasets, namely *LCA-Count* and *SpaM-4*. There are situations, however, where *SpaM-4* is superior to *LCA-Count*. If a leaf labelled with some reference sequence *S_i_* is far away from the other leaves in the reference tree, but the distance between a query read *q* and *S_i_* is small, then *LCA*-based methods would place *q* at the proximal branch of the node connecting *S_i_* and its neighbour in the tree. The query would, thus, be placed far away from *S_i_*. In contrast, our heuristic *SpaM-4* would be able to correctly place *q* near the leaf labelled with *S_i_*. Therefore, *SpaM-4* is the default version of our program; we advise the user to always use *App-SpaM* with the default settings.

In comparison to existing programs, besides *APPLES*, *App-SpaM* does not rely on a multiple sequence alignment of the reference sequences. This gives *App-SpaM* distinct advantages over existing, alignment-based programs: Calculating a reference MSA in a pre-processing step is time-consuming, and errors in the reference alignment can be a source of errors in the placement results. Moreover, if the homologies between the reference sequences are not co-linear, due to evolutionary events such as genome rearrangements, it is not possible to find a meaningful multiple alignment for them in the first place. This advantage of *App-SpaM* also implies that it does not require assembled reference sequences as input, but can be applied to taxa for which only unassembled reads are available. Genome assembly is still a non-trivial task (Padovani de Souza *et al.*, 2019; Sohn and Nam, 2018), and wrongly assembled reference sequences can be a source of errors in phylogenetic placement. Also, for a growing number of genomes, only unassembled reads are available, often with low sequencing depth (Coissac *et al.*, 2016; Dodsworth, 2015). For this, as a proof of concept, we performed phylogenetic placement on two datasets of viral and bacterial genomes of varying coverage, respectively. Here, the placement accuracy predominantly depends on the coverage of the reference sequences. In general, with unassembled references, the placement is not as accurate as when using assembled references but it approaches similar accuracy if the sequencing coverage is sufficiently high. However, the placement on unassembled references also comes at the cost of an increased variance of the placement accuracy: Here, accurate placement is only possible for those query reads where the corresponding homologous regions are also present in the ‘bags of reads’ of the references.

In this context, another certain difficulty for our *SpaM* approach can be caused by low-complexity regions in the input sequences, e.g. by long runs of ‘ATATATATAT’. If such runs would be present in both compared sequences, a large number of *SpaMs* with 100% matches at the don’t care positions would be found. For the viral and bacterial sequences used in this paper, low-complexity regions are not an issue. However, we would recommend to filter out low-complexity regions prior to running our software on eukaryotic sequences by using a software such as *RepeatMasker* (Smit and Green, 2015).

Another drawback of *App-SpaM*, as well as *APPLES*, compared to other programs, is their inability to describe the uncertainty of inferred placement locations. While *App-SpaM* only reports a single placement location for every query sequence, other programs can specify multiple placement locations for a single query read and assign weights to these locations according to their likelihood, so-called *likelihood-weight ratios*.

We used the benchmarking system *PEWO* for our evaluations to ensure clearly defined and reproducible evaluation workflows. A certain draw-back of *PEWO* is the fact that it simulates query reads by simply splitting the pruned reference sequences into segments of the desired read length; sequencing errors are not modelled. To evaluate our method under more realistic conditions, we performed additional test runs with simulated query reads obtained with the simulation software *ART* (Huang *et al.*, 2012), within the *PEWO* framework. Unlike *PEWO*, *ART* models sequencing errors. A conspicuous result of these test runs is that our method outperformed all other approaches substantially when we used reads simulated by the *ART* software (see Supplementary Section S4).

There are several possibilities to further improve *App-SpaM*. The placement heuristics that we implemented so far depend either on the number of spaced-word matches *or* on the estimated phylogenetic distances between a query read and the references. However, both sources of information may complement one another, so improved placement results might be obtained by combining both measures of distance and similarity, respectively. Such a placement method could also be used to express placement uncertainty similar to the likelihood-weight ratios used by *ML*-based methods. Hence, we are continuing to work on additional placement heuristics that use all available information to fully utilize the spaced-word matches approach for phylogenetic placement. Moreover, while the runtime of *App-SpaM* is already fast, the current implementation is not yet optimized for speed and memory efficiency and multiple strategies to further decrease the runtime are possible: All spaced words are held in main memory in a non-optimized data format instead of referencing the corresponding positions in the input sequences. This significantly increases the runtime and memory usage and can be improved by more efficient data handling. Given the test results shown in this study, *App-SpaM* should already be a useful tool for performing phylogenetic placement on large datasets and efforts should be made to further improve its accuracy and efficiency.

## Supplementary Material

vbab027_Supplementary_Data
