# Supplementary material for: App-SpaM: phylogenetic placement of short reads without sequence alignment
Source: Bioinform Adv. 2021 Oct 13;1(1):vbab027. doi: 10.1093/bioadv/vbab027 (PMC9710606; doi:10.1093/bioadv/vbab027)
Supplement: vbab027_Supplementary_Data [file vbab027_supplementary_data.pdf]

Supplementary Material for

bioRxiv 10.1101/2020.10.19.344986

Phylogenetic placement of short reads without sequence alignment

Matthias Blanke[1,3] and Burkhard Morgenstern[1,2,3]

[1]University of Göttingen, Institute of Microbiology and Genetics, Department of Bioinformatics,
[2]Campus-Institute Data Science (CIDAS),
Goldschmidtstr. 1, 37077 Göttingen, Germany
[3]International Max Planck Research School for Genome Science, Am Fassberg 11, 37077 Göttingen, Germany

September 27, 2021

Contents

1 Methods **3**
1.1 Inserting a Query Read into the Reference Tree . . . 3
1.2 Evaluation using PEWO . . . 4
1.3 Benchmark Datasets . . . 4
1.4 Evaluation Unassembled Reads: Own Pipeline . . . 5
1.5 Pattern Design . . . 6

2 Additional Results – *App-SpaM* **7**
2.1 *App-SpaM* Parameters . . . 7
2.2 Additional Results – *Pattern Robustness* . . . 10
2.3 Relation Between Difficulty of Pruning and Achieved Node Distance . . . 13

3 Detailed Results for Main Manuscript **20**
3.1 Boxplot Statistics . . . 20
3.2 Varying Read Lengths . . . 21
3.3 Accuracy with Different Parameters . . . 23
3.4 Memory Usage . . . 28
3.5 Run Time Large Showcase . . . 29

4 Additional Results – ART **30**

Overview

In the main paper, we describe a novel approach for *phylogenetic placement* of short reads called ***A**lignment-free **p**hylogenetic **p**lacement algorithm based on **Spa**ced-word **M**atches (App-SpaM)*. Our approach finds a suitable position for a query read sequence Q in a reference tree T. More precisely, for a given set of reference sequences and a binary rooted tree T with a one-to-one mapping between the sequences and the leaves of T, we describe different methods to find an edge e_Q of T where a query read Q can be inserted, based on our previously published *Filtered-Spaced Word Matches (FSWM)* approach [8], see also [7, 6, 13]. *App-SpaM* inserts a new node into e_Q, splitting e_Q into two new edges, and adds another new edge, connecting e_Q with a new leaf that is labelled with the query Q. The main paper describes methods to find a suitable edge e_Q, but does not explain how the lengths of the newly generated edges are defined.

The methods section of this *supplementary material* recapitulates our methods to find an edge e_Q where a query read Q is inserted into a reference tree T. In addition, we explain how the lengths of the newly generated edges are defined, see Subsec. 1.1. In the main paper, we use the *Placement Evaluation WOrkflows (PEWO)* [9] to evaluate the accuracy and run time of *App-SpaM* in most analyses. We discuss the parameters and accuracy metrics used for this evaluation in Subsec. 1.2. Additional information about the used data sets is given in Subsec. 1.3. However, the evaluations on unassembled references were not performed with PEWO because it does not support unassembled reference sequences. Instead, we used our own simplified evaluation pipeline which is described in Subsec. 1.4. We also present additional information about the pattern design in Subsec. 1.5.

This supplementary also contains additional results for *App-SpaM* and comprehensive reports for the accuracy of all programs to which we compared *App-SpaM* in our main paper – *RAPPAS* [10], *EPA* [3], *EPA-ng* [2], *Pplacer* [12], and *APPLES* [1] –, using a variety of parameter values. Here, Sec. 2 shows three additional results specifically for *App-SpaM*. First of all, additional results for the performance of different parameters in *App-SpaM* are given in Subsec. 2.1. Second, Subsec. 2.2 shows additional results for *App-SpaM* with respect to the choice of different binary patterns that are used for the spaced word matches. Third, additional plots for the relation between the 'difficulty' of pruning experiments and the accuracy of *App-SpaM* and all other tools are given in Subsec. 2.3.

Section 3 shows additional detailed information for the evaluations carried out in the main manuscript. This includes detailed statistics of the boxplots for the general accuracy evaluation in Subsec. 3.1, additional boxplots for different read lengths and their statistics in Subsec. 3.2, additional results for the accuracy of all programs when different parameters are used in Subsec. 3.3, a table for the memory requirements in Subsec. 3.4, and run time results for the large run time showcase on the *tara-3748* data set in Subsec. 3.5. Lastly, Sec. 4 shows additional results for the accuracy when query reads are simulated with the software *ART* [5] (in comparison to the simpler evaluation procedure used in PEWO).

1 Methods

1.1 Inserting a Query Read into the Reference Tree

For an edge e in an edge-weighted tree T, let $l(e)$ denote the length ('weight') of e. For a query sequence Q, we first select an edge e_Q in the reference tree T and insert a new internal node into this edge, thereby splitting e_Q into two new edges e_1 and e_2 with $l(e_1) + l(e_2) = l(e_Q)$. Then, we add a new leaf that is labelled with Q, together with a new edge e'_Q that connects this new leaf with the newly generated internal node. Finally, a length $l(e'_Q)$ is assigned to the newly generated edge e'_Q. To find a suitable edge e_Q for a query sequence Q, and to assign lengths to the newly generated edges, we first apply our approach *FSWM* to compare the Q with each reference sequence S. We are then using either the phylogenetic distances $d(Q, S)$ between Q and S as calculated by *FSWM*, or the number $s(Q, S)$ of spaced-word matches found by *FSWM*, with scores larger than some threshold t, between Q and S.

We implemented the following four approaches to find an edge e_Q where the query Q is inserted into the tree T:

MIN-DIST – In this approach, we first select the reference sequence S that minimizes the distance $d(Q, S)$ over all reference sequences, and we define e_Q to be the edge in T that is adjacent to the leaf labelled with S. If multiple references have the same smallest distance to Q, one of them is chosen randomly. As explained above, we split e_Q into two new edges e_1 and e_2. Let e_1 be the new edge that is adjacent to the leaf labelled with S. We distinguish the following two situations:

(A) If $d(Q, S)/2 < l(e_Q)$, the length of e_1 and e'_Q are set to $l(e'_Q) = l(e_1) = d(Q, S)/2$, and the length of e_2 is set to $l(e_Q) - l(e_1)$.

(B) If $d(Q, S)/2 \geq l(e)$, we set $l(e_1) = l(e_Q)$, $l(e_2) = 0$ and $l(e'_Q) = d(Q, S) - l(e_Q)$.

SpaM-COUNT – This works like the previous approach, but instead of selecting the reference sequence S that minimizes the distance to Q, we select the reference S that maximizes the number $s(Q, S)$ of spaced-word matches with score $> t$ between S and Q. The distances for the new edges are calculated in MIN-DIST.

LCA-DIST – Here, we identify the *two* reference sequences S_1 and S_2 with the lowest distances $d(Q, S_1)$ and $d(Q, S_2)$ to Q. Let v be the *lowest common ancestor* in T of the two leaves that are labelled with S_1 and S_2, respectively. The edge e_Q is then defined as the edge in T that connects v with its parental node. Let $l(S_1, v)$ be the sum of edge lengths between S_1 and v, and $l(S_2, v)$ accordingly. We define $\hat{d}(Q)$ as the average distance $\frac{d(Q,S_1)+d(Q,S_2)}{2}$ between Q and the two chosen references. We define $\hat{d}(v)$ as the average distance $\frac{l(S_1,v)+l(S_2,v)}{2}$ from the internal node v to the two chosen references in the tree T. To determine the new edge lengths of e_1 and e'_Q we distinguish three situations:

(A) If $\hat{d}(Q) < \hat{d}(v)$, we set $l(e_1) = 0$, $l(e_2) = l(e_Q)$, and the length of e'_Q is set to $l(e'_Q) = 0$.

(B) If $\hat{d}(v) < \hat{d}(Q) < \hat{d}(v) + 2 \cdot l(e_Q)$, the length of e_1 and e'_Q are set to $l(e_1) = l(e'_Q) = \frac{\hat{d}(Q)-\hat{d}(v)}{2}$, and $l(e_2)$ is set to $l(e_Q) - l(e_1)$.

(C) If $\hat{d}(Q) > \hat{d}(v) + 2 \cdot l(e_Q)$, then we set $l(e_1) = l(e_Q)$, $l(e_2) = 0$, and $l(e'_Q) = \hat{d}(Q) - \hat{d}(v) - l(e_Q)$.

LCA-COUNT – This is the same as the previous approach, but instead of using reference sequences S_1 and S_2 minimizing the distance with Q, we select the two references S_1 and S_2 with the maximal number of spaced-word matches to Q with scores larger than t. We then calculate distances for the newly generated edges as in LCA-DIST.

In addition to these four variants of *App-SpaM*, we used the distances $d(Q, S)$ calculated by *FSWM* as input for the program *APPLES* [1].

1.2 Evaluation using PEWO

For the accuracy evaluation we used the *Pruning-based ACcuracy evaluation (PAC)* workflow in *PEWO*. *PEWO* provides two accuracy measures to evaluate phylogenetic-placement methods, the *node distance (ND)* and the *expected node distance (e-ND)*. In short, *ND* is the number of nodes between the position where the query Q is placed into the tree T by a method that is to be evaluated, and the 'correct' position of Q, see [9] for details. Note that some methods assign to a query Q not one single position in T, but output several possible positions, that are weighted in some way. For likelihood-based methods, these weightings correspond to the calculated likelihood values normalized to 1 and are referred to as *likelihood-weight ratios*. In this case, *ND* measures only the distance between the *first* proposed placement – i.e. the one with the largest weight – and the 'correct' position of Q. As an alternative, *PEWO* offers the so-called *e-ND* metric, to evaluate multiple weighted placement positions. Here, for a single query, the number of nodes between *every* proposed placement and the 'correct' branch is taken into account with respect to its according weight.

The current version of *App-SpaM* outputs for each query Q a *single* position in T. The same is true for *APPLES*. Therefore, we used the *ND* metric for the evaluations shown in the paper. Because placement uncertainty can be represented with the *e-ND* metric, *e-ND* is typically smaller than the *ND* metric. We show comprehensive results with all *ND* and *e-ND* values for programs across all data sets in Subsec. 3.3. These results also include the accuracy across all parameters that were tested during the evaluation. In general, while *App-SpaM* cannot compute *likelihood* values, it could theoretically also derive multiple placement positions for a single query Q based on the number of spaced-word matches to all references and the calculated distances. With our proposed placement heuristics, this might be especially reasonable when several reference sequences have a very similar number of spaced word matches or phylogenetic distance to a given query Q. However, specifying such multiple locations and weighting them appropriately requires extensive additional tests that will take us some time to perform.

In the main paper, we showed one accuracy value (*ND*) for all programs and data sets. This value is for the default parameters of each program; these parameters are shown in Table 1.

App-SpaM	RAPPAS	APPLES	EPA-ng	EPA	Pplacer
$w = 12$<br>m: EXP-4	$k = 8$, omega: 1.5<br>reduction: 0.99	method: FM<br>criteria: MLSE	heuristic: 1<br>$g = 0.999$	$g = 0.1$	$ms = 6, sb = 3$<br>$mp = 40$

Table 1: Parameter choices for the summarized results shown in the paper.

1.3 Benchmark Datasets

Each data set in *PEWO* [9] consists of a multiple sequence alignment of the reference sequences and a phylogenetic tree comprising exactly these references. Reads are always simulated automatically by *PEWO* by splitting pruned reference sequences into shorter reads (with exception to the unassembled reference sequence simulations, see Subsec. 1.4). The table given in the main manuscript contains the number and length of the reference sequences in the data sets from *PEWO*, together with the length of the simulated reads that we used. The origin of these data sets is given below. With regard to ultrametricity, we also report the difference in distance between the root and the closest/farthest leaf. Let d_c be the distance between the root and the closest leaf and d_f the distance between the root and the farthest leaf, we report $\frac{d_f}{d_c}$.

bac-150 A set of 16S RNA sequences of 150 taxa across multiple bacterial orders with $\frac{d_f}{d_c} = 129.9$. Taken from the examples section of *PEWO*, available at:
https://github.com/phylo42/PEWO

vir-104 Complete viral genomes of 104 *HIV* strains that include most subtypes with $\frac{d_f}{d_c} = 8.67$. Taken from the examples of *PEWO*, available at:
https://github.com/phylo42/PEWO

neotrop-512 This data set comprises 512 sequences of 16S rDNA and was published in [11] with $\frac{d_f}{d_c} = 17.86$. It was used for the evaluation in *EPA-ng* [2]. In comparison to the evaluation performed in *EPA-ng*, we only use the references and not the provided query sequences. It is freely available at:
https://datadryad.org/stash/dataset/doi:10.5061/dryad.kb505nc

tara-3748 This 16S rDNA data set comprises with 3748 the most references sequences and was published in [15] with $\frac{d_f}{d_c} = 72.68$. It was used in *EPA-ng* and is available at:
https://datadryad.org/stash/dataset/doi:10.5061/dryad.kb505nc

bv-797 16S rDNA reference data set from [14] with $\frac{d_f}{d_c} = 341.42$. It was used in *EPA-ng* and is available at:
https://datadryad.org/stash/dataset/doi:10.5061/dryad.kb505nc

epa-218 One of the data sets used for the evaluation of EPA [3] consisting of 218 sequences of the small sub-unit rRNA with $\frac{d_f}{d_c} = 37.76$. It was kindly supplied by Alexandros Stamatakis, as were the next two data sets. We are not aware of any availability online for these three data sets.

epa-628 One of the data sets used for the evaluation of EPA [3] consisting of 628 fungal DNA sequences with $\frac{d_f}{d_c} = 21.85$.

epa-714 One of the data sets used for the evaluation of EPA [3] consisting of 714 sequences of the small sub-unit rRNA with $\frac{d_f}{d_c} = 13.89$.

CPU-652 The resources evaluation data set used in the example section of *PEWO*, online available at:
https://github.com/phylo42/PEWO

CPU-512 Same data set as *neotrop-512*.

wol-43 Data set of 43 whole genomes of different *Wolbachia* species with $\frac{d_f}{d_c} = 2.12$.

1.4 Evaluation Unassembled Reads: Own Pipeline

All evaluations, except with non-assembled reference sequences, were carried out with the *PEWO* framework to ensure reliability, correctness, and easy reproducibility. However, *PEWO* has limitations: First, it does not simulate sequencing errors for the query reads. We added this feature for additional tests, see Sec. 4. Second, it is limited in its applicability and does not support other input types for the reference sequences than a multiple sequence alignment of the references. In order to evaluate the performance of *App-SpaM* on unassembled reference sequences, we implemented a simpler version of the *PEWO* PAC-workflow as follows: First, we simulated reads of a defined coverage from the input reference sequences with the program *ART* [5]. We used coverages of 4, 2, 1, 0.5, 0.25, 0.125, 0.0625, 0.03125. The resulting 'bags of reads' constitute the reference sequences for the experiments. Then, we use a leave-one-out procedure already used by *pplacer* [12]. A single reference (bag of reads) is pruned out from the reference data set. The remaining bags of reads and the accordingly pruned backbone tree are used as the reference data

set. All reads from the pruned reference are placed onto the pruned backbone tree and the accuracy is measured with the ND metric. The average ND of all queries constitutes the overall ND for this pruning event. Then, the procedure is repeated once for every reference sequence. The average over all pruning events for a given coverage is the recorded as the accuracy for this coverage. The procedure is repeated for all coverages.

Evolutionary Models: Jukes-Cantor

Currently, *App-SpaM* uses the Jukes-Cantor model to estimate the number of substitutions that happened based on the calculated hamming distance from the don't care positions of all filtered spaced word matches. Preliminary experiments showed little to no improvement of the accuracy when using more sophisticated models such as K80 or K81. However, too little tests were carried out to strongly support this observation, neither did we test other parameter-rich ones like GTR. In general, we are planning to test the influence of more complicated evolutionary models when using the filtered spaced-word matches approach, but this is not scope of this work.

1.5 Pattern Design

The default parameters for the pattern set used in *App-SpaM* is $|\mathcal{P}| = 1$ (a single pattern is used), with weight $w = 12$ and 32 don't care positions. The weight $w = 12$ has already worked well in *FSMW* [8] and *Read-SpaM* [6], and we see the same for *App-SpaM*. The influence of using more patterns simultaneously, of using different randomly optimized pattern sets, and the weight is shown in several figures in this supplementary, specifically Fig. 1, Fig. 2, Fig. 3, Fig. 4, Fig. 5. The number of don't care positions is more complicated to choose based on the use case: *FSWM* uses 100 don't care positions to compare whole genomes. Here, a large number of don't care positions helps to differentiate between the random background peak and the homologous peak of spaced-word matches. In *Read-SpaM* the default number of don't care positions is reduced to 60. This choice was made due to the short reads that are compared: A larger number of don't care positions results in a lower number of possible spaced word matches. As an example for a read length of 150: A pattern length of 112 as in FSWM results in only 39 possible spaced-word matches for a given read; reducing the pattern length to 72 already doubles the number of possible spaced word matches to 79. In practice, the increased number of spaced-word matches per read showed improved results. In *App-SpaM*, the number of don't care positions was decreased again to 32. Preliminary results showed that the results are rather stable for patterns of these lengths. This comes with the trade-off that background and homologous peaks can potentially overlap more strongly. However, based on these preliminary results, we made the design decision to store all don't care positions in a single 64 bit integer. This comes with large simplifications in the implementation that yield faster computations as a result. On the negative side, the number of don't care positions cannot be raised above 32, even if the results were better. It is also possible to use multiple patterns within *App-SpaM*. The sets of patterns are generated with the *rasbhari* [4] software that minimizes the variance of pattern-based matches between queries and references by evaluating the *overlap complexity* of the patterns in the set. For this, *rasbhari* uses a hill-climbing method to iteratively improve the pattern set, we refer to the paper for more details.

2 Additional Results – ***App-SpaM***

App-SpaM has 5 different placement heuristics, as well as the ability to forward calculated distances to *APPLES* to perform the placement. Additionally, the weight w of the used pattern(s) could potentially influence the placement accuracy. Generally, we strongly recommend to use *App-SpaM* with default values; that is the heuristic SpaM-4 with a single pattern of weight 12. In all our evaluations these settings resulted in robust and accurate results. We noticed some instances, specifically when using unassembled references, where lower weights yielded improved results. We also performed an extensive evaluation to examine the accuracy of *App-SpaM* with respect to the five heuristics and different weights on a number of unrelated data sets. Here, however, we show for two data sets (*bac-150* and *hiv-104*) how the placement accuracy varies for pattern weights of $w \in \{8, 12, 16\}$, and for all 5 heuristics with $X \in \{2, 4\}$ for SpaM-X as well as *App-SpaM* distances in *APPLES* in the following. Similar results, in a simplified form, for all data sets are also shown in Subsec. 3.3.

2.1 ***App-SpaM*** Parameters

bac-150 data set, read length 15

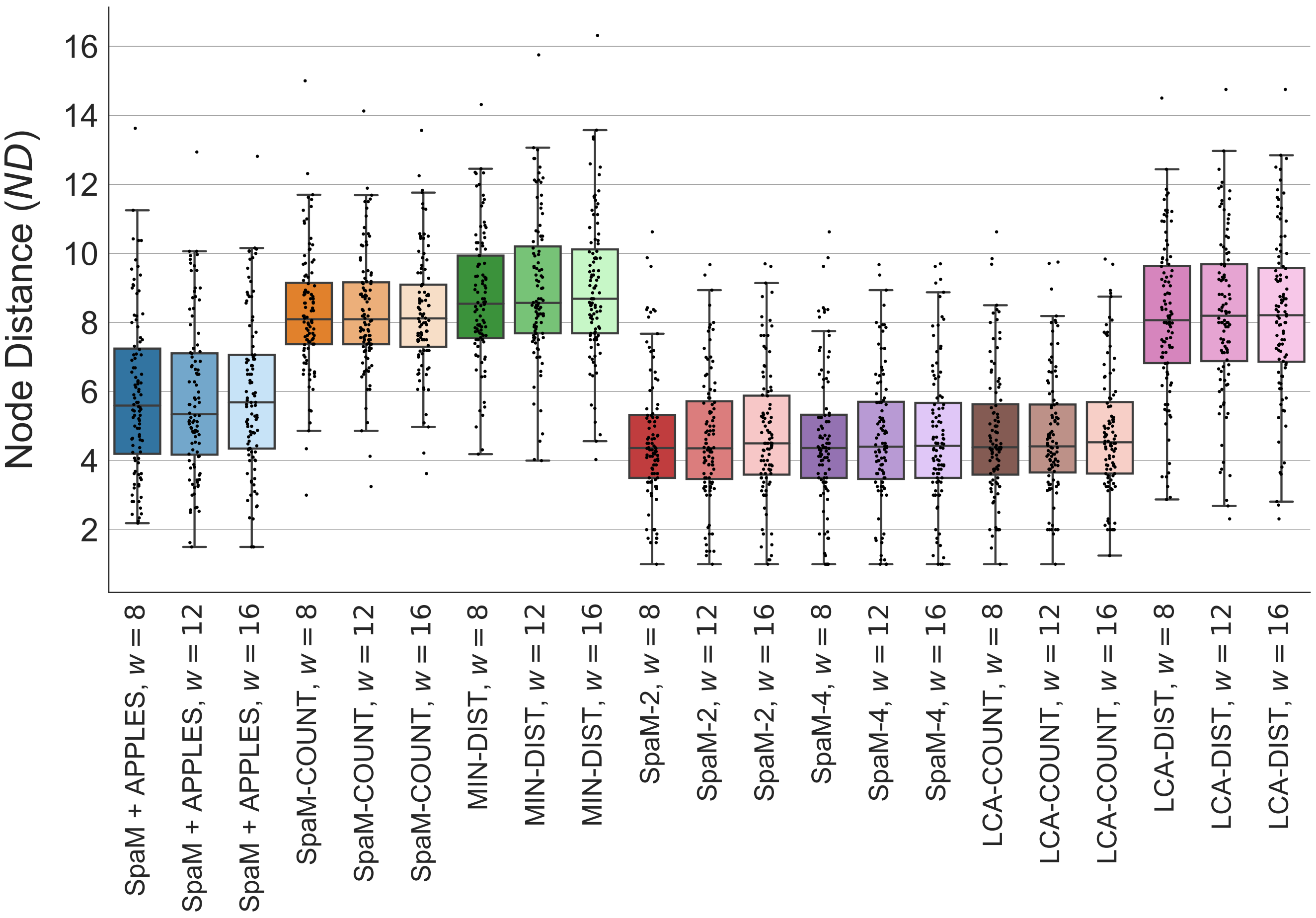

Figure 1: Performance of *App-SpaM* on the *bac-150* data set for read lengths of 150 with $n = 100$ prunings. The heuristics and pattern weights were modified according to the annotated x-axis. For each heuristic, three different boxes exist for $w \in \{8, 10, 12\}$ (colors always with decreasing luminosities from $w = 8$ to $w = 12$).

hiv-104 data set, read length 150

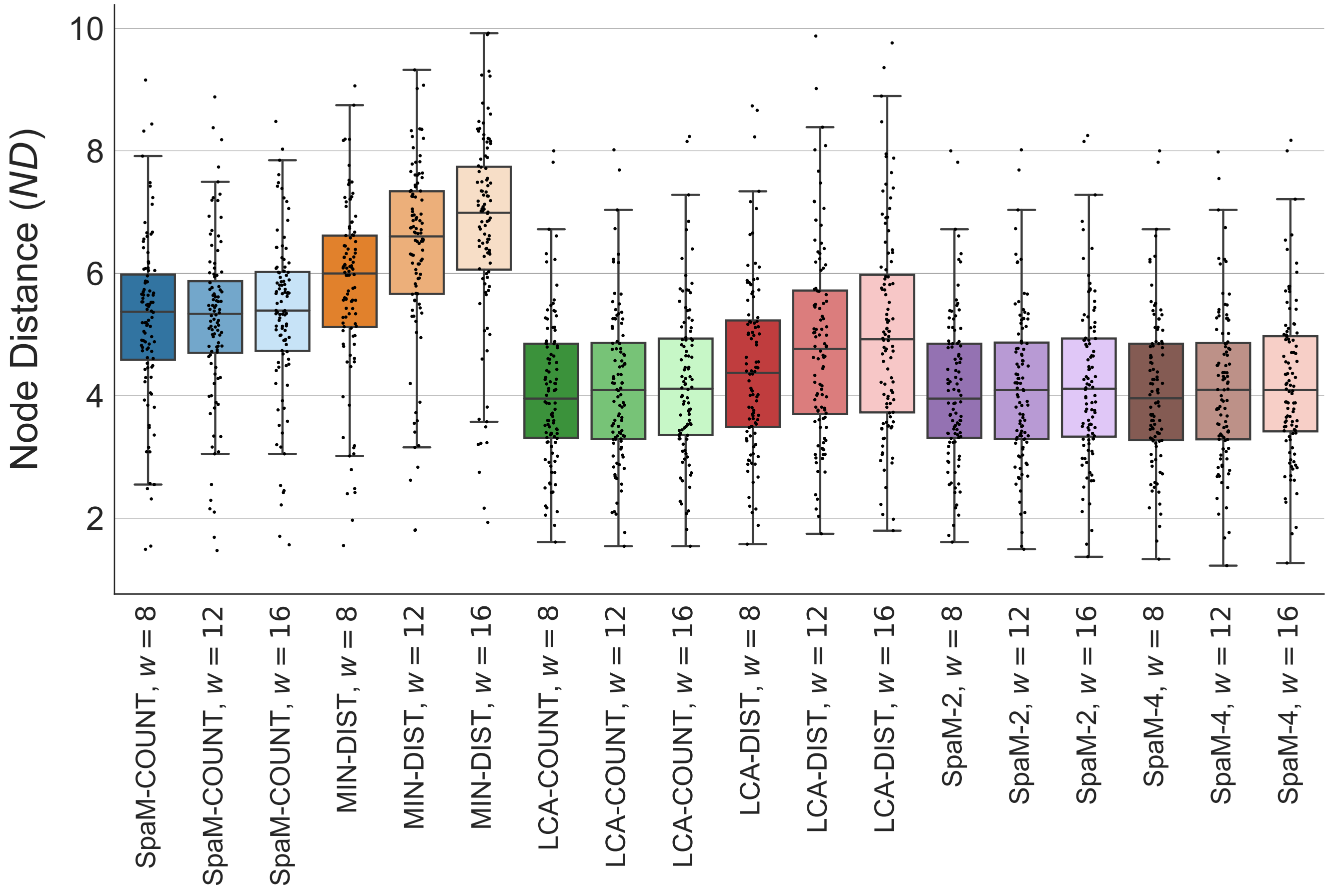

Figure 2: Performance of *App-SpaM* on the *hiv-104* data set for read lengths of 150 with $n = 100$ prunings. The heuristics and pattern weights were modified according to the annotated x-axis. For each heuristic, three different boxes exist for $w \in \{8, 10, 12\}$ (colors always with decreasing luminosities from $w = 8$ to $w = 12$).

hiv-104 data set, read length 500

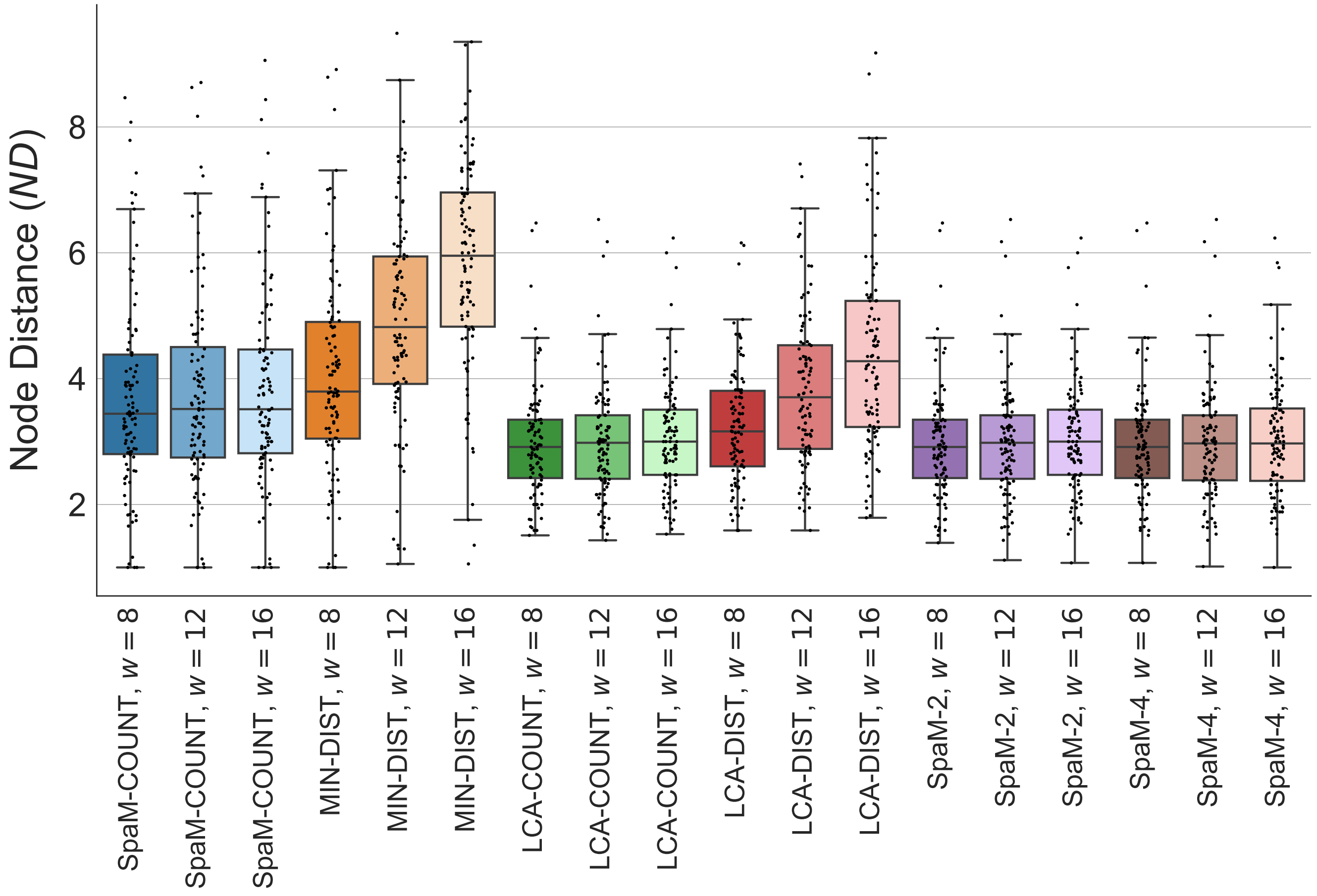

Figure 3: Performance of *App-SpaM* on the *hiv-104* data set for read lengths of 500 with $n = 100$ prunings. The heuristics and pattern weights were modified according to the annotated x-axis. For each heuristic, three different boxes exist for $w \in \{8, 10, 12\}$ (colors always with decreasing luminosities from $w = 8$ to $w = 12$).

2.2 Additional Results – ***Pattern Robustness***

App-SpaM uses a set of patterns, denoted as $\mathcal{P}$. By default, the size of this pattern set is $|\mathcal{P}| = 1$, however, it is possible to use multiple patterns. This feature exists mainly for our internal tests. Generally, $\mathcal{P}$ for a given weight w and number of don't care positions is chosen by optimizing the overlap complexity for the pattern set with the software *rasbhari* [4]. We evaluated the robustness of *App-SpaM* with respect to different generated pattern sets and the number of such patterns that are simultaneously used. Here shown are the results for the data set *bac-150* and the data set *bv-797* and the two heuristics *LCA-COUNT* and *SpaM-4* of *App-SpaM*. Generally, we do not see any pronounced influence between either the number of patterns and the accuracy, or between different patterns and the accuracy.

bac-150 data set

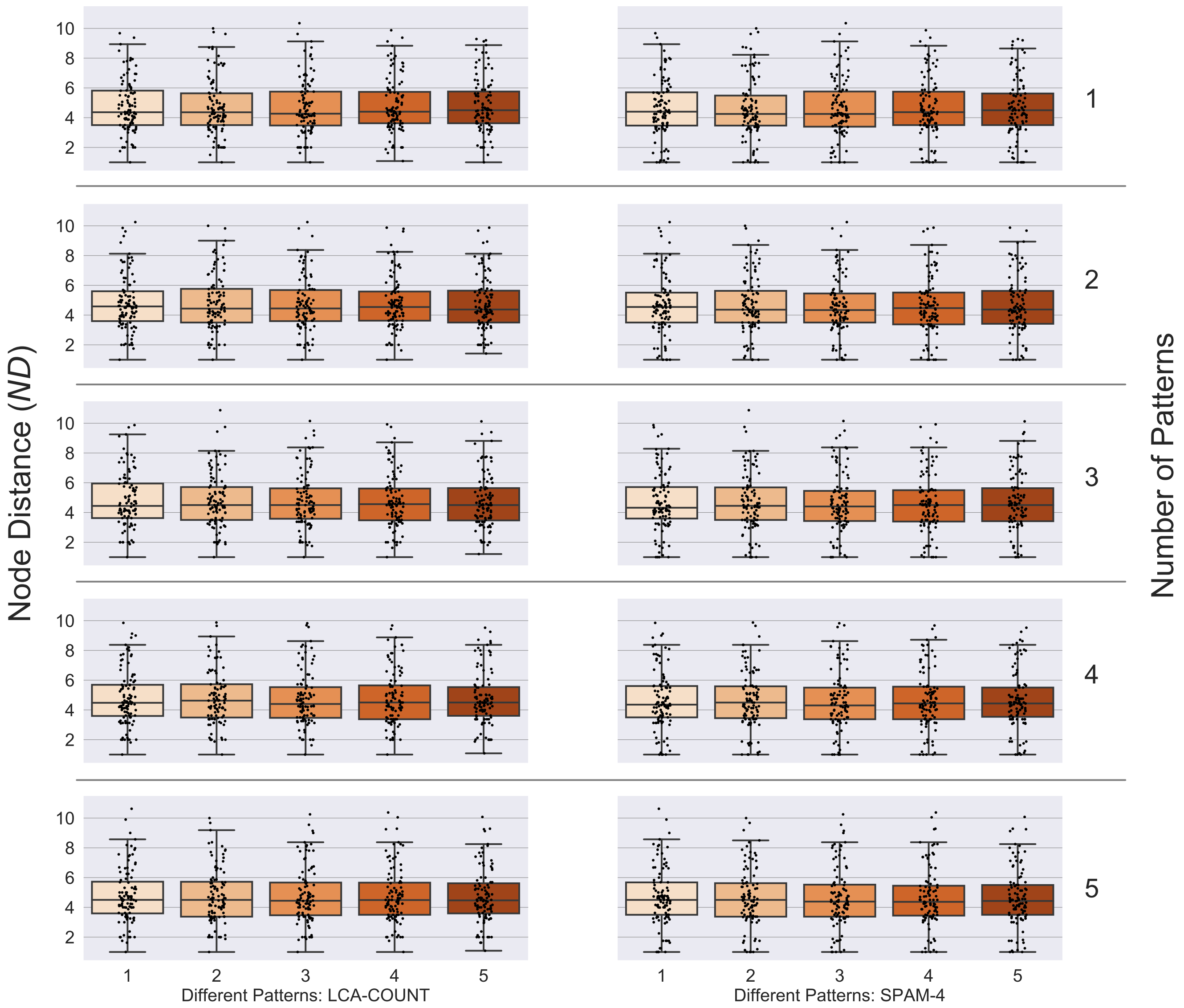

Figure 4: Performance of *App-SpaM* on the *bac-150* data set for a varying number of patterns (between 1 and 5), and 2 different heuristics. The two overall columns differ in the used placement heuristic: *LCA-COUNT* (left), *SpaM-4* (right). Every row, consisting of two plots each, shows the results for a different number of patterns that are used simultaneously (1 pattern at the top row up until 5 patterns at the bottom row). Each single setting (gray patch) shows the results for 5 different pattern sets (light orange to dark orange, each pattern set optimized with a different random seed as input) of the specified lengths over $n = 100$ prunings each. Every black dot corresponds to a single pruning event. The y-Axis on each plot corresponds to the accuracy of the pruning experiment. Y-scales on all plots are chosen identically. Read lengths were always fixed at 150.

bv-797 data set

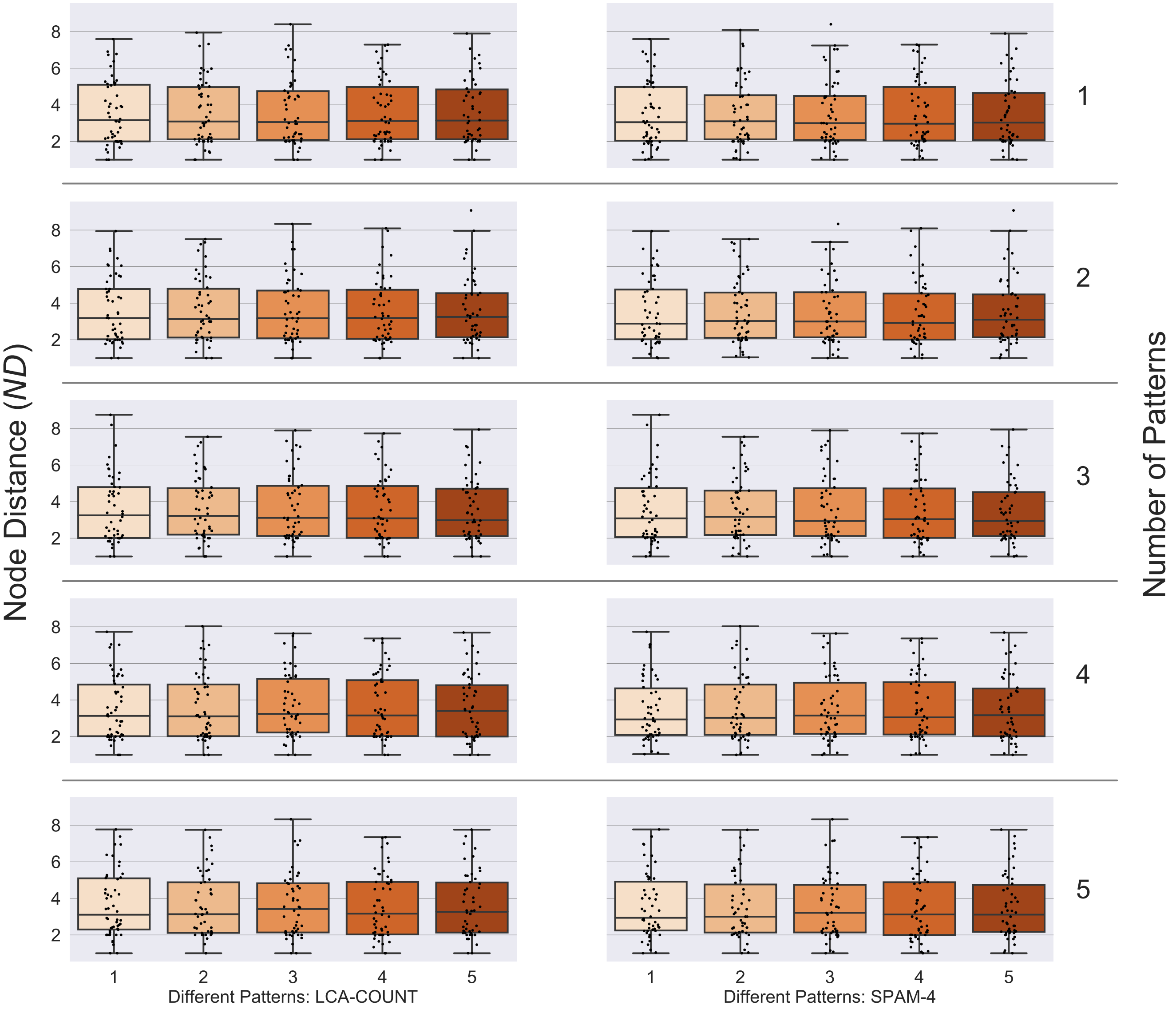

Figure 5: Performance of *App-SpaM* on the *bv-797* data set for a varying number of patterns (between 1 and 5), and 2 different heuristics. The two overall columns differ in the used placement heuristic: *LCA-COUNT* (left), *SpaM-4* (right). Every row, consisting of two plots each, shows the results for a different number of patterns (1 pattern at the top row up until 5 patterns at the bottom row). Each single setting (gray patch) shows the results for 5 different pattern sets (light orange to dark orange, each pattern set optimized with a different random seed as input) of the specified lengths over $n = 50$ prunings each. Every black dot corresponds to a single pruning event. The y-Axis on each plot corresponds to the accuracy of the pruning experiment. Y-scales on all plots are chosen identically. Read lengths were always fixed at 150.

2.3 Relation Between Difficulty of Pruning and Achieved Node Distance

In PEWO, for every pruning event a random node within the tree is chosen and all references below this node are removed. Thus, resulting prunings can vary: from only a single pruned leave, to a few references, to large or very large clades of the references tree. Thus, a case can be made, that prunings vary with respect to their 'difficulty'. However, it is unclear how the 'difficulty' can be described reasonably, or, in other words, which property of a pruning makes it more or less difficult. One possibility could be, that prunings where the correct branch is located deep within the tree, i.e. more closely towards the root (and possibly no closely related reference remains in the tree), are more complex. However, not only the size of the pruned tree could be an indication of the 'difficulty' of a pruning, but also how far away the next reference sequences are from the pruned sequences within the tree. Here, we look at two measures for the difficulty of a pruning: The first proxy for the pruning difficulty is, thus, the difference in branch lengths between the unpruned and pruned reference tree. A large difference in branch lengths indicates that long branches or many sequences were pruned. Short branch length differences indicate that few and/or short branches were pruned. Second, we look at the *height* of the correct placement branch within the tree. Here, the height for the correct branch is defined as the number of nodes on the longest path towards any leaf below (including the leaf node). Thus, the minimal height of 1 indicates that the correct placement branch is directly above a leaf, while large numbers indicate that the correct placement branch is located more towards the root of the tree. We performed experiments to examine the relation between these two measures for pruning 'difficulty' and the achieved placement accuracy of all programs on three data sets: *bac-150*, *neotrop-512*, and *bv-797*. We also report correlation coefficients and p-values for the Spearman-correlation the accuracy of placements and between both measures, respectively.

With a significance level of $\alpha = 0.05$ we observe the following: For the first measure (difference in branch lengths), *App-SpaM* shows a significant positive correlation for all three data sets. This correlation is also present for all other programs on data set *neotrop-512* and for all programs except *RAPPAS* and *APPLES* on data set *bv-797*. For *bac-150*, only *EPA* shows the positive correlation. For the second measure, for two data sets, correlations between height of correct branch and accuracy are not significant for all programs. For the last data set (*bv-797*) positive correlations are significant for all programs except *APPLES* and *EPA*. However, for all experiments note the limited sample size of 100 prunings. In general, we expect to observe a correlation between a measure that represent the 'difficulty' of a pruning and the achieved accuracy on the prunings. However, what exactly constitutes as a 'difficult' pruning can also depend on the used placement software, as different input data sets might pose different demands on the software.

bac-150 data set

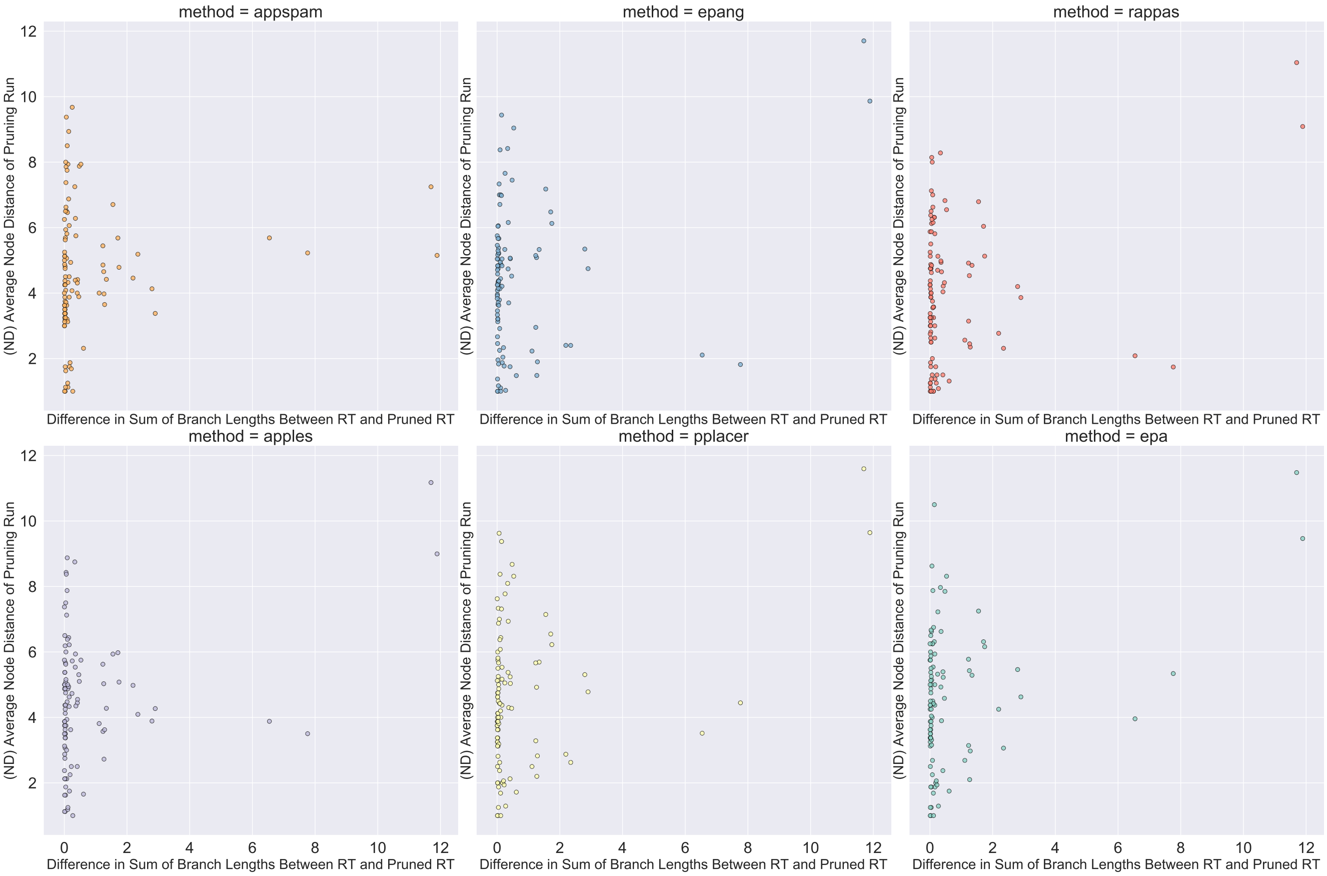

Figure 6: Relation between the difference in branch lengths of pruning and ground truth (x-axis) and performance of programs (y-axis). Every dot corresponds to a single pruning event and shows the average node distance of the programs, respectively. The x-axis on each plot corresponds to difference in branch lengths between original and pruned tree; we regard this as a proxy for the difficulty of the pruning experiment. Read lengths were always fixed at 150. Spearman correlation coefficient and p-value are given for all programs in the form *[software name] (correlation coefficient, p-value)* in the following: *App-SpaM* (0.25, 0.012), *EPA-ng* (0.13, 0.2), *RAPPAS* (0.105, 0.3), *APPLES* (0.167, 0.097), *PPlacer* (0.195, 0.0523), *EPA* (0.203, 0.042).

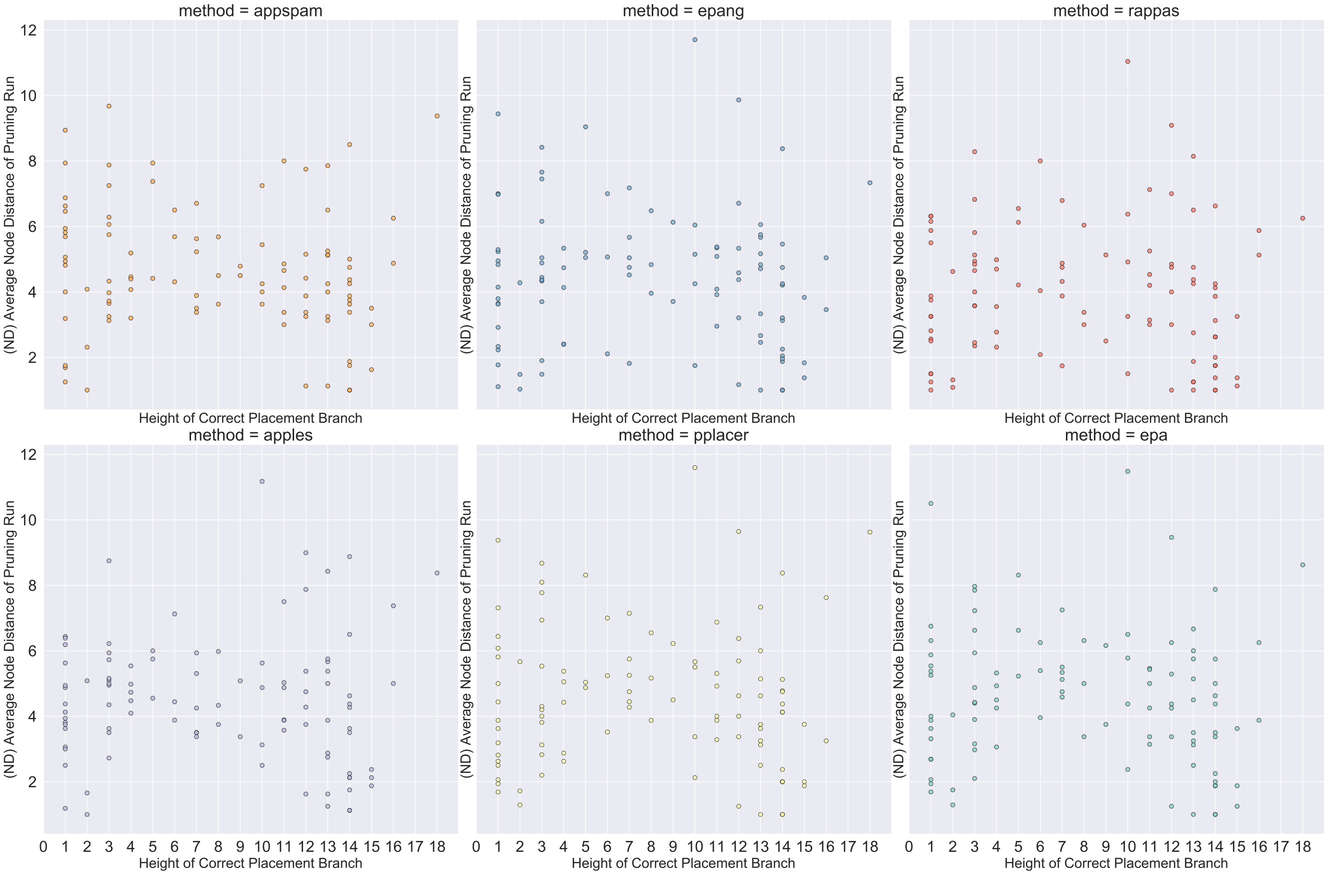

Figure 7: Relation between height of correct placement branch of prunings (x-axis) and accuracy of programs (y-axis). Every dot corresponds to a single pruning event and shows the average node distance of the programs, respectively. The x-axis on each plot corresponds to the height of the correct placement branch in each pruning run. Read lengths were always fixed at 150. Spearman correlation coefficient and p-value are given for all programs in the form *[software name] (correlation coefficient, p-value)* in the following: *App-SpaM* (-0.183, 0.0678), *EPA-ng* (-0.091, 0.37), *RAPPAS* (-0.081, 0.425), *APPLES* (-0.096, 0.341), *PPlacer* (-0.078, 0.442), *EPA* (-0.113, 0.261).

neotrop-512 data set

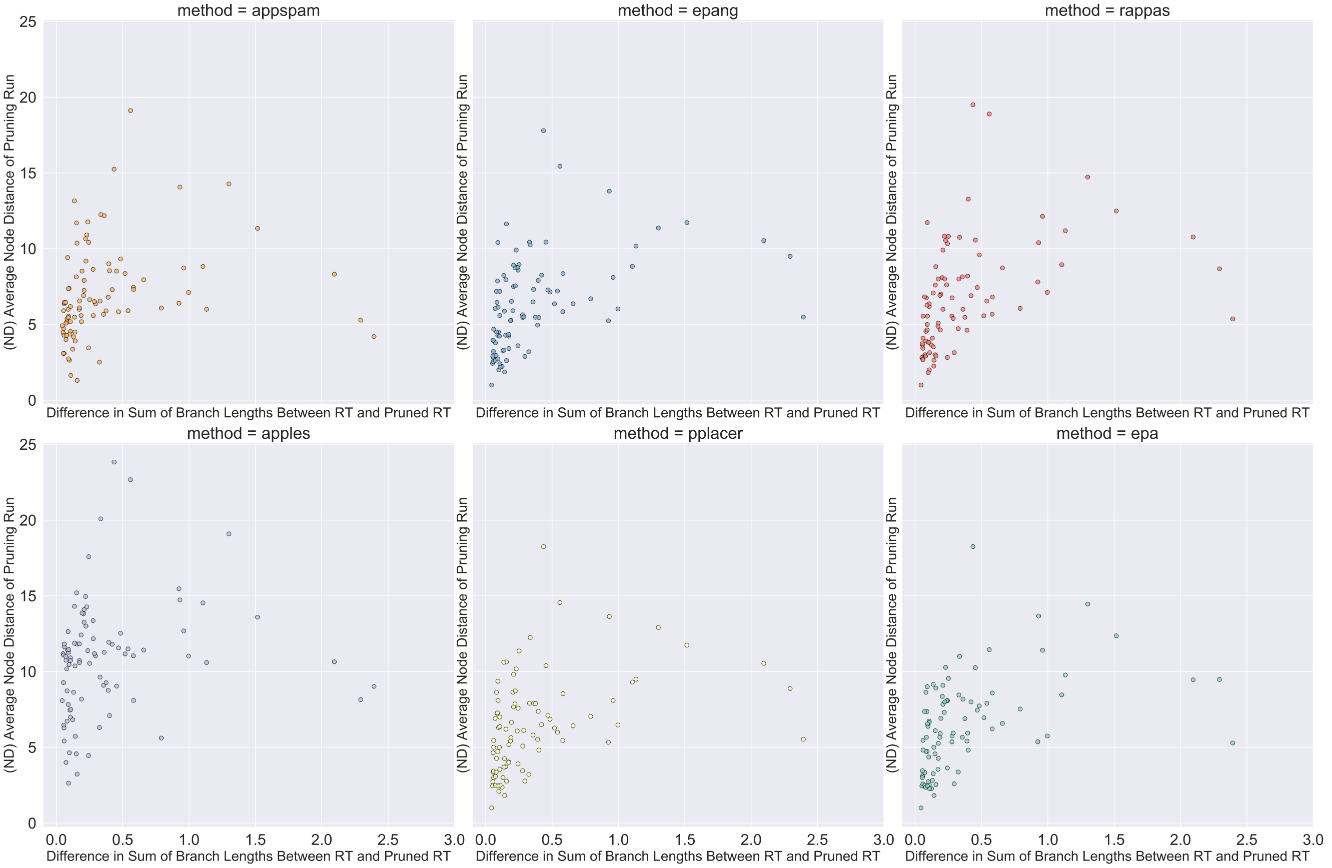

Figure 8: Relation between the difference in branch lengths of pruning and ground truth (x-axis) and performance of programs (y-axis). Every dot corresponds to a single pruning event and shows the average node distance of the programs, respectively. The x-axis on each plot corresponds to difference in branch lengths between original and pruned tree; we regard this as a proxy for the difficulty of the pruning experiment. Read lengths were always fixed at 150. Spearman correlation coefficient and p-value are given for all programs in the form *[software name] (correlation coefficient, p-value)* in the following: *App-SpaM* (0.535, $9.91 \cdot 10^{-9}$), *EPA-ng* (0.67, $2.524 \cdot 10^{-14}$), *RAPPAS* (0.696, $8.678 \cdot 10^{-16}$), *APPLES* (0.329, 0.00082), *PPlacer* (0.629, $2.421 \cdot 10^{-12}$), *EPA* (0.675, $1.407 \cdot 10^{-14}$).

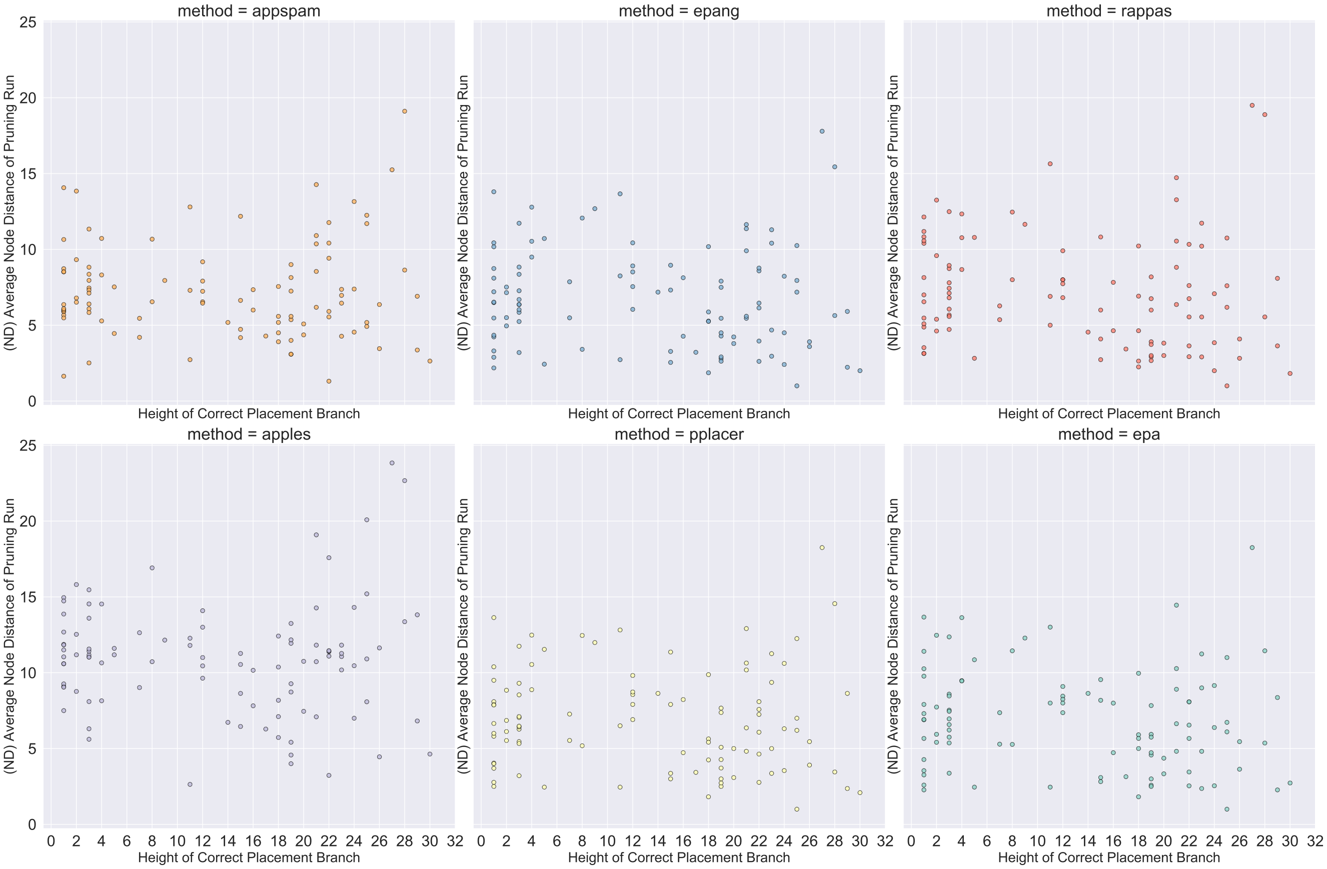

Figure 9: Relation between height of correct placement branch of prunings (x-axis) and accuracy of programs (y-axis). Every dot corresponds to a single pruning event and shows the average node distance of the programs, respectively. The x-axis on each plot corresponds to the height of the correct placement branch in each pruning run. Read lengths were always fixed at 150. Spearman correlation coefficient and p-value are given for all programs in the form *[software name] (correlation coefficient, p-value)* in the following: *App-SpaM* (-0.047, 0.64), *EPA-ng* (-0.124, 0.217), *RAPPAS* (-0.183, 0.068), *APPLES* (-0.046, 0.651), *PPlacer* (-0.104, 0.303), *EPA* (-0.142, 0.16).

bv-797 data set

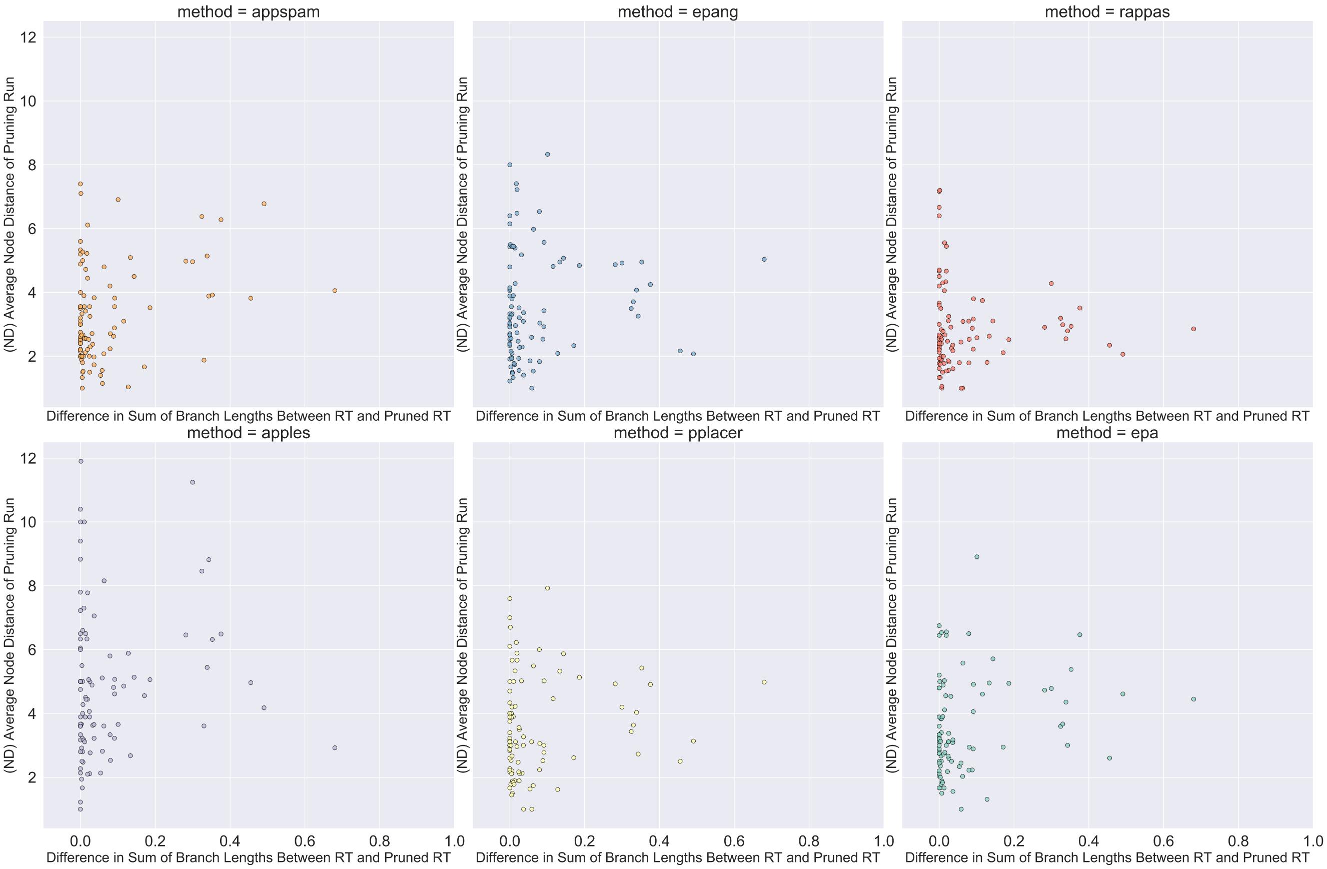

Figure 10: Relation between the difference in branch lengths of pruning and ground truth (x-axis) and performance of programs (y-axis). Every dot corresponds to a single pruning event and shows the average node distance of the programs, respectively. The x-axis on each plot corresponds to difference in branch lengths between original and pruned tree; we regard this as a proxy for the difficulty of the pruning experiment. Read lengths were always fixed at 150. Spearman correlation coefficient and p-value are given for all programs in the form *[software name] (correlation coefficient, p-value)* in the following: *App-SpaM* (0.271, 0.0056), *EPA-ng* (0.21, 0.036), *RAPPAS* (0.024, 0.81), *APPLES* (0.109, 0.28), *PPlacer* (0.203, 0.043), *EPA* (0.322, 0.001).

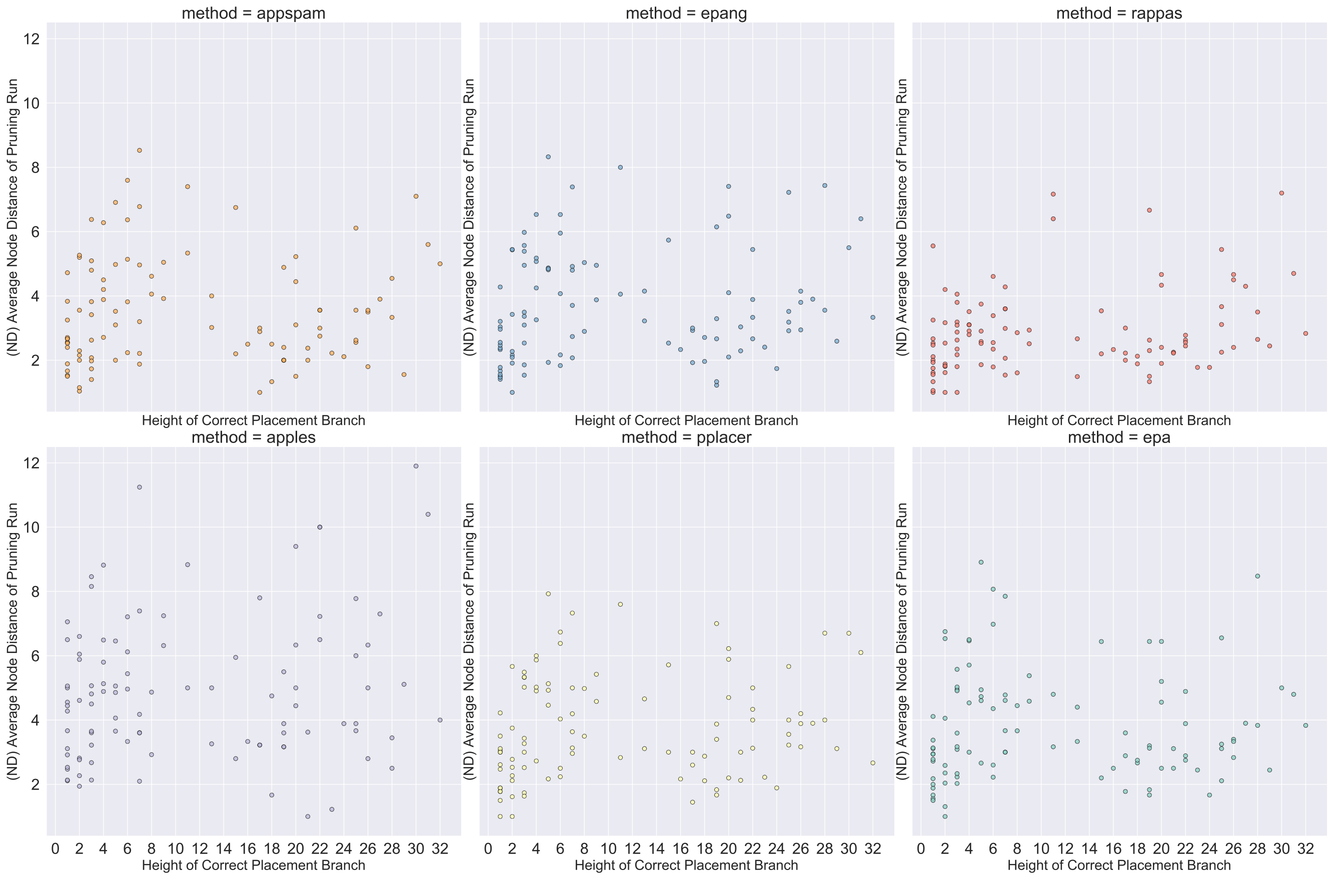

Figure 11: Relation between height of correct placement branch of prunings (x-axis) and accuracy of programs (y-axis). Every dot corresponds to a single pruning event and shows the average node distance of the programs, respectively. The x-axis on each plot corresponds to the height of the correct placement branch in each pruning run. Read lengths were always fixed at 150. Spearman correlation coefficient and p-value are given for all programs in the form *[software name] (correlation coefficient, p-value)* in the following: *App-SpaM* (0.129, 0.0203), *EPA-ng* (0.238, 0.017), *RAPPAS* (0.273, 0.006), *APPLES* (0.174, 0.083), *PPlacer* (0.263, 0.008), *EPA* (0.160, 0.111).

3 Detailed Results for Main Manuscript

Here, additional detailed results from the figures in the main manuscript are given. Subsection 3.1 shows detailed statistics of the box plots of the manuscript. Additional figures and detailed tables of experiments with different read lengths are shown in Subsec. 3.2. Lastly, Subsec. 3.3 has additional figure for all programs with different parameters and both metrics, ND and e-ND.

3.1 Boxplot Statistics

The main manuscript shows results of the accuracy of all six programs (appspam, pplacer, epa, epang, rappas, apples) on eight data sets as box plots in a single figure. The following tables show the corresponding exact values of the box plots (sample size (fixed at $n = 100$), mean, standard deviation (std), minimal (min) value, maximal (max) value, and quartiles) for all programs and data sets. For every data set, the method that has the lowest average values according to these tables is highlighted with a red star in the corresponding figure.

bac-150

	appspam	pplacer	epa	epang	rappas	apples
count	100.000	100.000	100.000	100.000	100.000	100.000
mean	4.651	4.572	4.578	4.414	3.998	4.492
std	1.937	2.148	2.074	2.131	2.071	1.929
min	1.000	1.000	1.000	1.000	1.000	1.000
25	3.470	3.125	3.227	2.854	2.487	3.469
50	4.403	4.400	4.463	4.408	3.938	4.375
75	5.703	5.673	5.757	5.336	5.126	5.558
max	9.675	11.596	11.480	11.704	11.040	11.175

hiv-104

	appspam	pplacer	epa	epang	rappas	apples
count	100.000	100.000	100.000	100.000	100.000	100.000
mean	4.132	3.908	3.834	3.688	3.731	4.235
std	1.253	0.967	0.943	0.848	0.940	1.145
min	1.226	1.226	1.191	1.121	1.600	1.294
25	3.288	3.261	3.200	3.155	3.046	3.459
50	4.098	3.874	3.757	3.646	3.710	4.149
75	4.861	4.367	4.275	4.183	4.298	4.949
max	7.983	7.323	7.613	6.151	6.781	8.047

neotrop-512

	appspam	pplacer	epa	epang	rappas	apples
count	100.000	100.000	100.000	100.000	100.000	100.000
mean	7.233	6.872	6.865	6.756	7.119	10.892
std	3.141	3.329	3.329	3.343	3.685	3.728
min	1.308	1.000	1.000	1.000	1.000	2.636
25	5.258	4.198	4.519	4.229	4.091	8.705
50	6.545	6.450	6.607	6.364	6.750	11.056
75	8.568	8.756	8.705	8.747	9.668	12.445
max	19.111	18.250	18.250	17.792	19.500	23.833

tara-3728

	appspam	pplacer	epa	epang	rappas	apples
count	100.000	100.000	100.000	100.000	100.000	100.000
mean	9.424	10.078	10.356	11.908	9.933	21.948
std	6.059	6.129	6.614	7.525	6.886	10.923
min	1.500	1.875	1.750	1.531	1.250	5.333
25	5.216	5.891	5.609	6.701	5.486	14.861
50	8.423	8.611	9.222	10.948	7.944	19.470
75	11.439	12.647	13.066	14.968	12.561	26.153
max	39.444	36.778	42.556	51.917	38.000	58.333

bv-797

	appspam	pplacer	epa	epang	rappas	apples
count	100.000	100.000	100.000	100.000	100.000	100.000
mean	3.507	3.735	3.772	3.687	2.844	4.937
std	1.678	1.626	1.698	1.730	1.274	2.256
min	1.000	1.000	1.000	1.000	1.000	1.000
25	2.209	2.516	2.566	2.333	1.983	3.250
50	3.099	3.334	3.225	3.275	2.552	4.681
75	4.640	4.986	4.785	4.927	3.284	6.320
max	8.529	7.927	8.909	8.327	7.200	11.900

epa-218

	appspam	pplacer	epa	epang	rappas	apples
count	100.000	100.000	100.000	100.000	100.000	100.000
mean	6.202	5.889	5.815	5.547	5.484	6.618
std	2.862	3.026	2.991	2.811	2.550	2.841
min	1.700	1.100	1.100	1.500	1.444	1.100
25	4.386	3.889	3.764	3.505	3.693	4.969
50	5.950	5.285	5.000	5.072	5.150	6.667
75	7.162	7.350	7.343	7.148	7.339	8.077
max	18.316	17.737	19.000	16.222	14.370	16.778

epa-628

	appspam	pplacer	epa	epang	rappas	apples
count	100.000	100.000	100.000	100.000	100.000	100.000
mean	5.434	6.511	6.470	6.180	6.422	8.612
std	3.086	3.230	3.326	3.213	3.670	3.696
min	1.000	1.700	1.800	1.367	1.000	2.478
25	3.200	4.000	3.800	3.800	3.546	5.481
50	4.800	6.233	5.883	5.533	5.944	8.800
75	6.864	7.998	8.050	7.474	8.007	10.664
max	18.000	16.000	16.500	17.000	19.500	17.500

epa-714

	appspam	pplacer	epa	epang	rappas	apples
count	100.000	100.000	100.000	100.000	100.000	100.000
mean	4.617	4.315	4.391	4.105	3.973	5.451
std	1.864	1.835	1.824	1.856	1.883	2.128
min	1.000	1.125	1.286	1.083	1.000	1.143
25	3.421	3.000	3.094	2.876	2.729	3.983
50	4.250	3.969	4.016	3.667	3.469	5.179
75	5.413	5.375	5.528	5.048	4.929	6.696
max	11.714	10.571	11.000	11.333	10.571	11.786

3.2 Varying Read Lengths

Due to space constraints, the main manuscript shows only a simplified version of the performance of the algorithms for different lengths of the query reads. Here, additional box plots for the results for different read lengths are given, as well as their exact statistics in the tables below. All y-axes show ND.

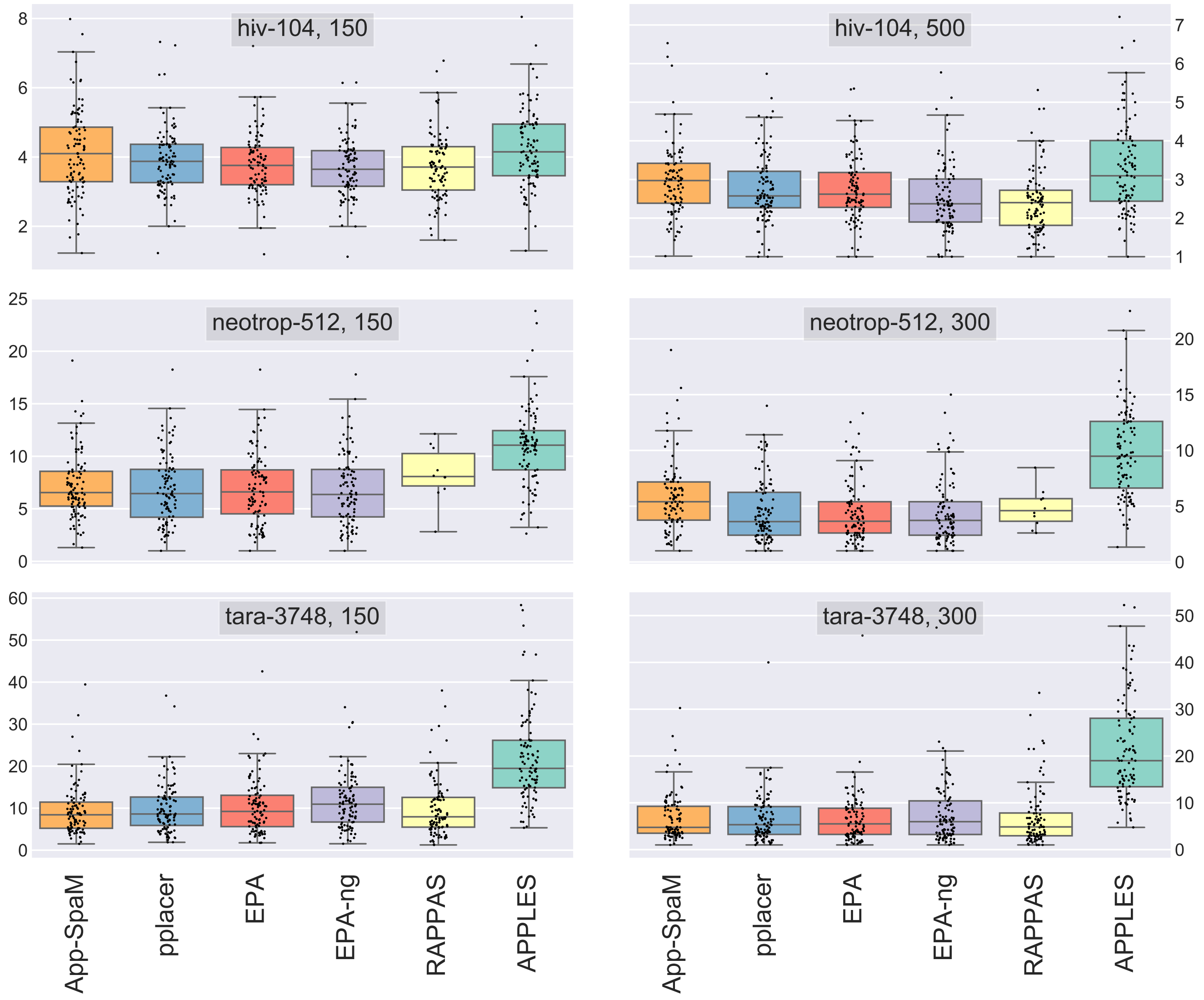

Figure 12: Average node distance for query reads of length 150, 300, 500 *bp* as shown, respectively. All y-axes show the average node distance (ND). Every row is one of three different data sets. Every column is for a different read lengths (either 150, 300, or 500, see figure titles). Every black dot corresponds to the average ND of a single pruning experiment. Every box corresponds to the statistics over $n = 100$ pruning events.

hiv-104

	$epang_r$150	$epang_r$500	epa_r150	epa_r500	$rappas_r$150	$rappas_r$500	$pplacer_r$150	$pplacer_r$500	$apples_r$150	$apples_r$500	$appspam_r$150	$appspam_r$500
count	100.000	100.000	100.000	100.000	100.000	100.000	100.000	100.000	100.000	100.000	100.000	100.000
mean	3.688	2.520	3.834	2.767	3.731	2.430	3.908	2.771	4.235	3.298	4.132	2.988
std	0.848	0.885	0.943	0.851	0.940	0.834	0.967	0.883	1.145	1.213	1.253	0.949
min	1.121	1.000	1.191	1.000	1.600	1.000	1.226	1.000	1.294	1.000	1.226	1.014
25	3.155	1.899	3.200	2.277	3.046	1.812	3.261	2.268	3.459	2.437	3.288	2.385
50	3.646	2.370	3.757	2.620	3.710	2.400	3.874	2.574	4.149	3.095	4.098	2.971
75	4.183	3.010	4.275	3.181	4.298	2.721	4.367	3.211	4.949	4.007	4.861	3.418
max	6.151	5.772	7.613	5.353	6.781	5.316	7.323	5.737	8.047	7.211	7.983	6.529

neotrop-512

	$epang_r$150	$epang_r$300	epa_r150	epa_r300	$rappas_r$150	$rappas_r$300	$pplacer_r$150	$pplacer_r$300	$apples_r$150	$apples_r$300	$appspam_r$150	$appspam_r$300
count	100.000	100.000	100.000	100.000	10.000	10.000	100.000	100.000	100.000	100.000	100.000	100.000
mean	6.756	4.452	6.865	4.472	8.319	4.822	6.872	4.565	10.892	9.785	7.233	5.861
std	3.343	2.875	3.329	2.755	2.680	1.768	3.329	2.784	3.728	3.993	3.141	3.208
min	1.000	1.000	1.000	1.000	2.818	2.600	1.000	1.000	2.636	1.333	1.308	1.000
25	4.229	2.400	4.519	2.596	7.175	3.650	4.198	2.400	8.705	6.625	5.258	3.750
50	6.364	3.725	6.607	3.650	8.071	4.600	6.450	3.618	11.056	9.481	6.545	5.400
75	8.747	5.400	8.705	5.400	10.260	5.675	8.756	6.241	12.445	12.600	8.568	7.173
max	17.792	15.000	18.250	13.333	12.136	8.457	18.250	14.000	23.833	22.500	19.111	19.000

tara-3748

	$epang_r$150	$epang_r$300	epa_r150	epa_r300	$rappas_r$150	$rappas_r$300	$pplacer_r$150	$pplacer_r$300	$apples_r$150	$apples_r$300	$appspam_r$150	$appspam_r$300
count	100.000	100.000	100.000	100.000	100.000	100.000	100.000	100.000	100.000	100.000	100.000	100.000
mean	11.908	7.610	10.356	6.618	9.933	6.761	10.078	6.689	21.948	21.303	9.424	6.719
std	7.525	6.546	6.614	5.525	6.886	6.164	6.129	5.298	10.923	10.814	6.059	4.974
min	1.531	1.000	1.750	1.000	1.250	1.000	1.875	1.000	5.333	4.750	1.500	1.000
25	6.701	3.234	5.609	3.250	5.486	2.958	5.891	3.250	14.861	13.438	5.216	3.500
50	10.948	5.969	9.222	5.500	7.944	4.850	8.611	5.321	19.470	19.000	8.423	4.750
75	14.968	10.411	13.066	8.837	12.561	7.821	12.647	9.191	26.153	28.062	11.439	9.250
max	51.917	47.438	42.556	45.750	38.000	33.500	36.778	40.000	58.333	52.250	39.444	30.250

3.3 Accuracy with Different Parameters

The accuracy results in the main manuscript show the average node distance of every program for a specific combination of program parameters (the default ones), see Subsec. 1.2. We also only used ND throughout the paper to ensure comparability between all programs with respect to the same accuracy metric. Here, we show additional results for all programs with different parameters, as well as the e-ND for all programs that support it. Every bar corresponds to the average accuracy across all pruning events. The accuracy for a single pruning event is given as the average over all placed query reads. All parameters on the x-axis labels are abbreviated in accordance with *PEWO*:

App-SpaM	
mode	placement heuristics (mindist, spamcount, lcadist, spamx)
w	weight of patterns
pattern	number of patterns to use
APPLES	
meth	least squares method to use
crit	placement criterion (least squares phylogenetic placement, minimum evolution, or hybrid)
RAPPAS	
k	size of phylo-k-mers in data base, larger values are more accurate but slower
o	probability threshold for RAPPAS
red	reduction: gap/non-gap ratio above which site of alignment is ignored
ar	software for ancestral state reconstruction
PPlacer	uses a two-step placement heuristic called the "baseball"-heuristic
ms	max-strikes
sb	strike-box
mp	max-pitches
EPA-ng	
h	used heuristic by EPA-ng, 1 is fastest, 2 slower but more accurate
EPA	
g	proportion of top scoring branch for which full optimization is computed

Table 2: Parameter abbreviations used in the x-axes labels of all following plots.

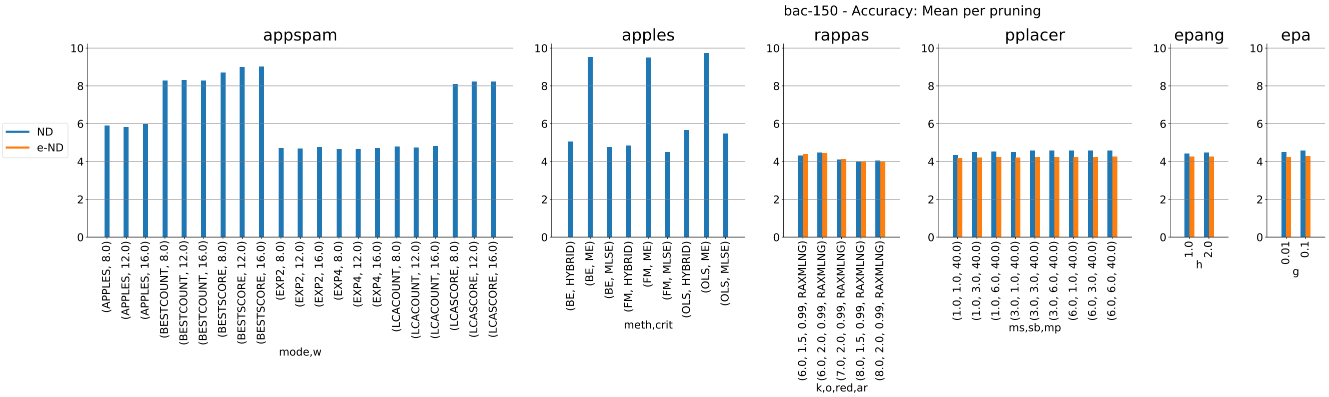

Figure 13: Accuracy results for specified data set for different program parameters. ND always shown in blue, e-ND in orange. For programs where the accuracy was very similar across all tested parameters, only a small subset of the parameter space is shown. Query read lengths fixed at 150.

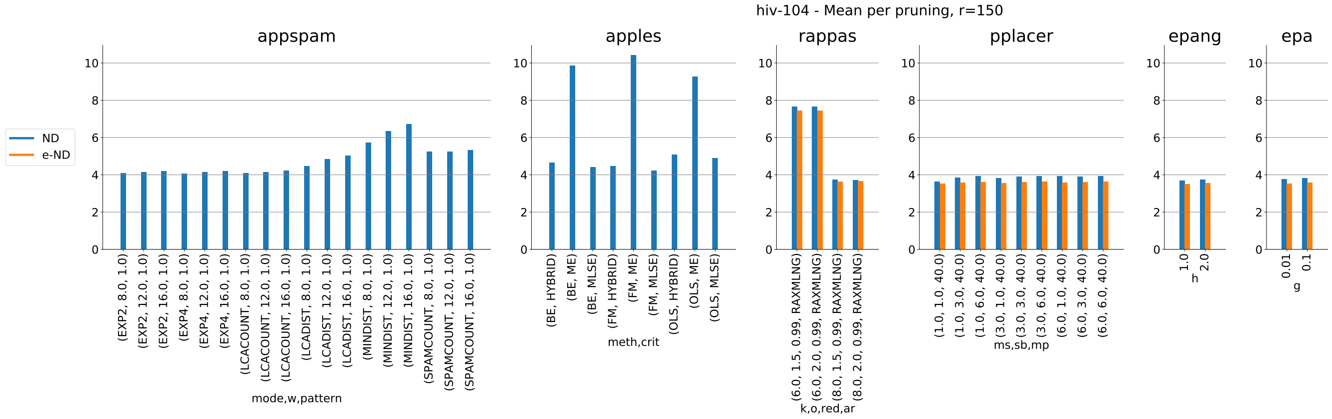

Figure 14: Accuracy results for specified data set for different program parameters. ND always shown in blue, e-ND in orange. For programs where the accuracy was very similar across all tested parameters, only a small subset of the parameter space is shown. Query read lengths fixed at 150.

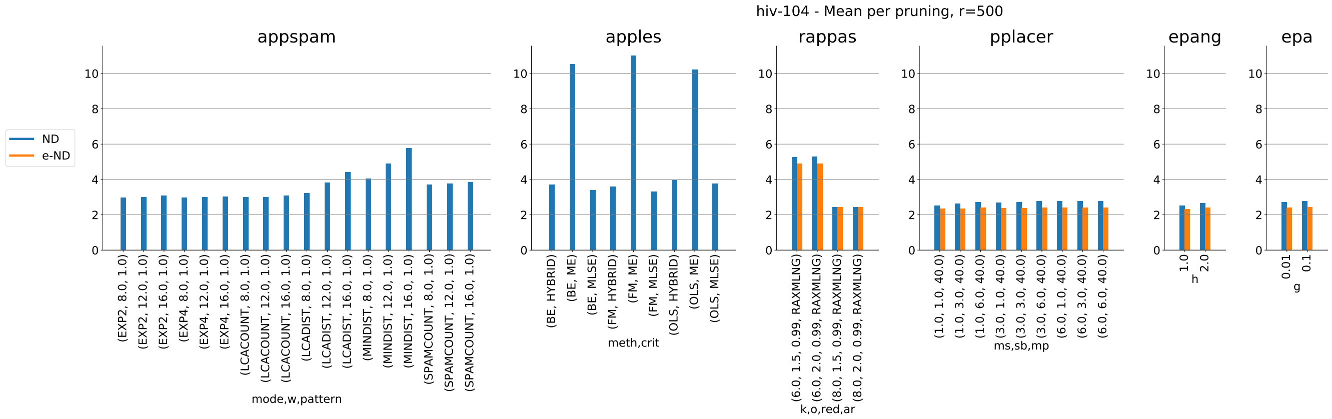

Figure 15: Accuracy results for specified data set for different program parameters. ND always shown in blue, e-ND in orange. For programs where the accuracy was very similar across all tested parameters, only a small subset of the parameter space is shown. Query read lengths fixed at 500.

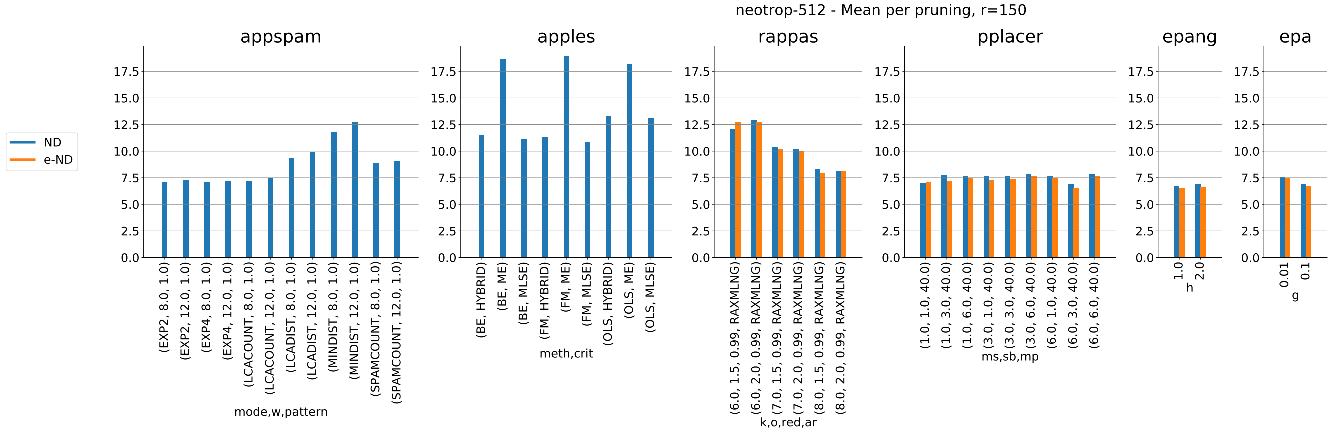

Figure 16: Accuracy results for specified data set for different program parameters. ND always shown in blue, e-ND in orange. For programs where the accuracy was very similar across all tested parameters, only a small subset of the parameter space is shown. Query read lengths fixed at 150.

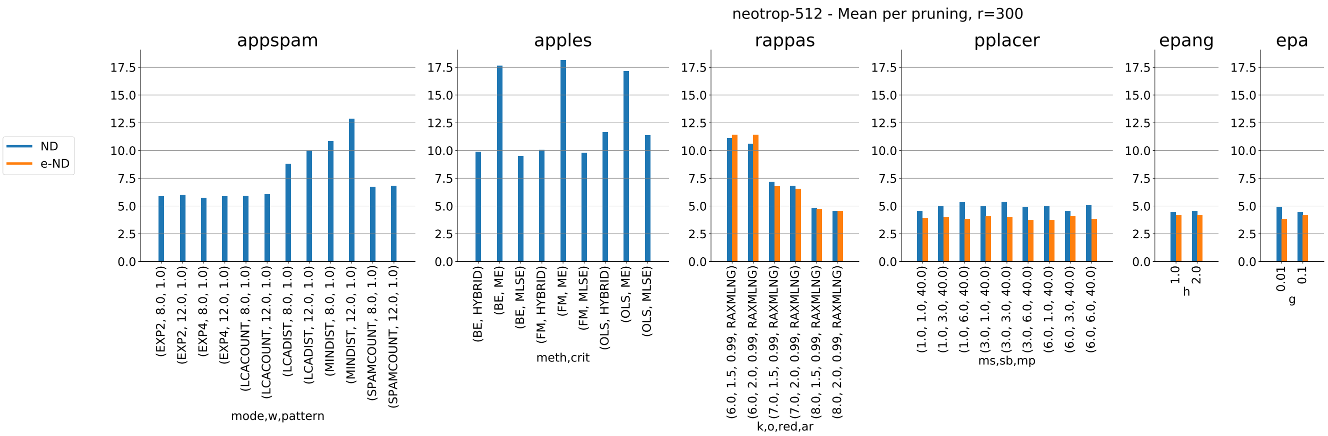

Figure 17: Accuracy results for specified data set for different program parameters. ND always shown in blue, e-ND in orange. For programs where the accuracy was very similar across all tested parameters, only a small subset of the parameter space is shown. Query read lengths fixed at 300.

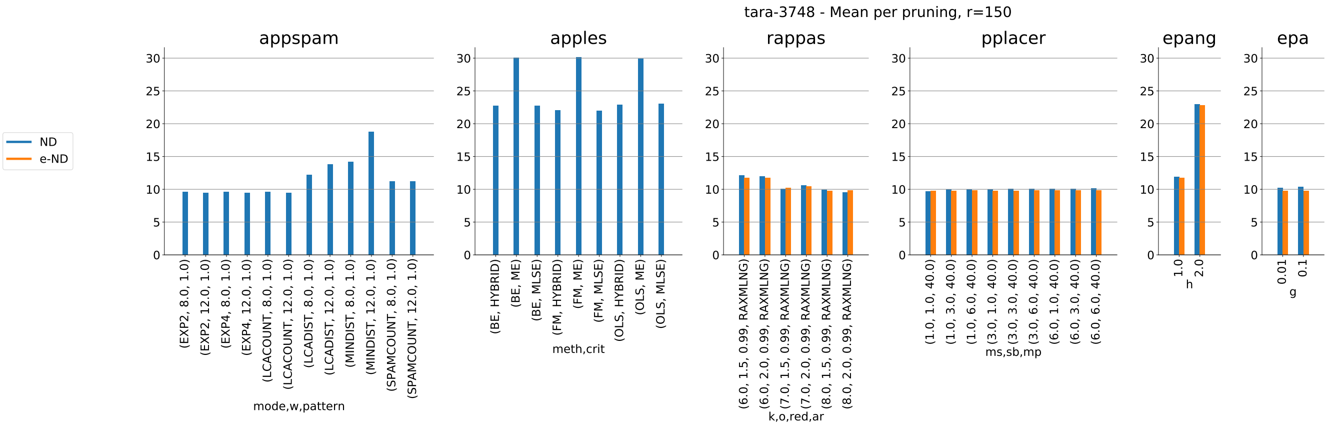

Figure 18: Accuracy results for specified data set for different program parameters. ND always shown in blue, e-ND in orange. For programs where the accuracy was very similar across all tested parameters, only a small subset of the parameter space is shown. Query read lengths fixed at 150.

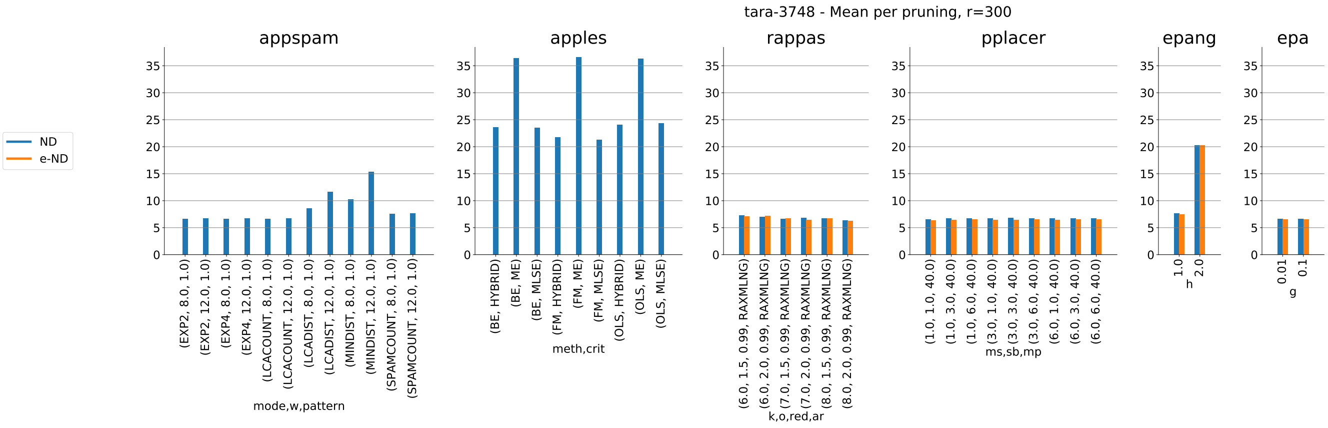

Figure 19: Accuracy results for specified data set for different program parameters. ND always shown in blue, e-ND in orange. For programs where the accuracy was very similar across all tested parameters, only a small subset of the parameter space is shown. Query read lengths fixed at 300.

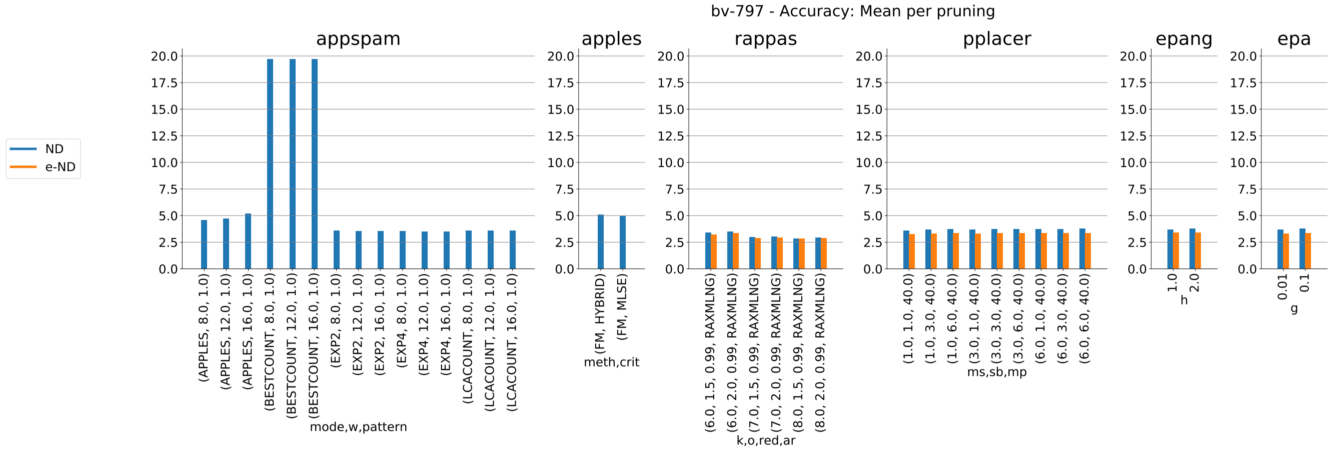

Figure 20: Accuracy results for specified data set for different program parameters. ND always shown in blue, e-ND in orange. For programs where the accuracy was very similar across all tested parameters, only a small subset of the parameter space is shown. Query read lengths fixed at 150.

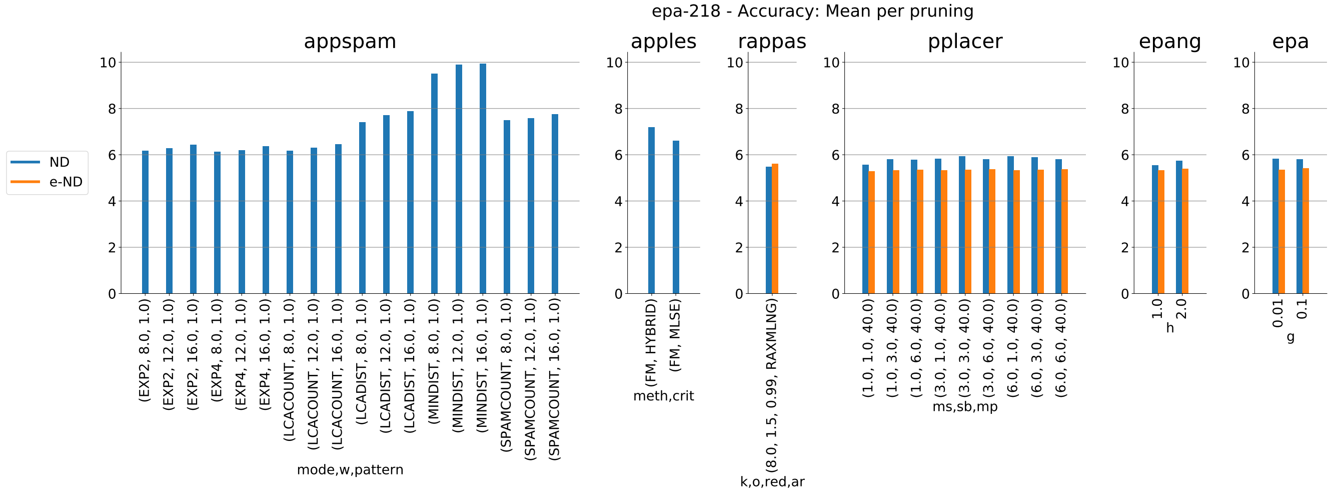

Figure 21: Accuracy results for specified data set for different program parameters. ND always shown in blue, e-ND in orange. For programs where the accuracy was very similar across all tested parameters, only a small subset of the parameter space is shown. Query read lengths fixed at 150.

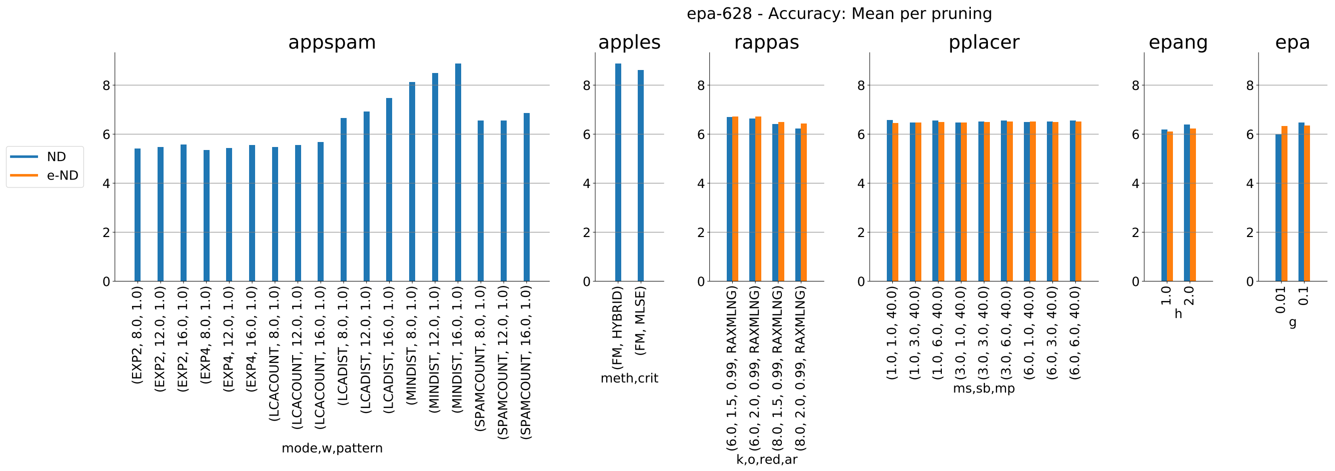

Figure 22: Accuracy results for specified data set for different program parameters. ND always shown in blue, e-ND in orange. For programs where the accuracy was very similar across all tested parameters, only a small subset of the parameter space is shown. Query read lengths fixed at 150.

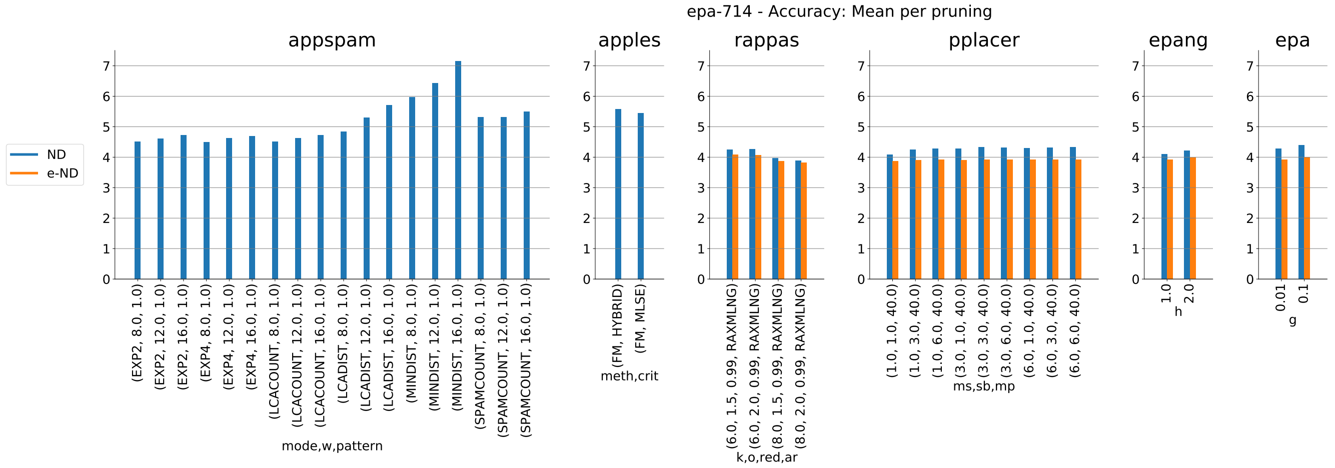

Figure 23: Accuracy results for specified data set for different program parameters. ND always shown in blue, e-ND in orange. For programs where the accuracy was very similar across all tested parameters, only a small subset of the parameter space is shown. Query read lengths fixed at 150.

3.4 Memory Usage

We performed a run time and memory usage evaluation using *PEWO*'s resources workflow. The reported run times are shown in the main manuscript. Here, we show the results for the memory usage for the two data sets *CPU-652* and *CPU-512*. All shown results are taken from the *pss-max* column of the results reported by PEWO. PSS measures the *proportional set size* which gives a reasonable estimate of the total memory usage of the system (shared libraries between processes are only counted once in comparison to RSS). For more detailed information we refer to the extensive *PEWO* manual on Github.

Memory usage of *App-SpaM* is mainly dominated by two data structures whose size directly depends on the number and lengths of input references and queries. First, the design of the spaced words: Each spaced word is saved in 28 bytes. This includes all base pairs at match and mismatch positions, as well as its originating sequence and position within the sequence. Thus, for input files that encode every symbol in a single byte, *App-SpaM's* memory footprint to save all spaced words can be estimated at 28 times the size of input files. Here, only the input of the references is relevant, since queries are read and processed in small batches. Second, a structure that saves statistics (the number of spaced words, mismatches, etc.) between all references and the currently handled queries. With n input references and m simultaneously handled queries, this structure needs roughly $16 \cdot n \cdot m$ bytes.

For short reference sequences, such as single marker genes, the second structure will dominate the memory footprint of *App-SpaM*. For long reference sequences the storage of the spaced words dominates the overall memory usage.

	App-SpaM		RAPPAS		APPLES	EPA-ng	EPA	pplacer
	$w = 12$	$w = 16$	$k = 6$	$k = 8$				
CPU-652								
preproc.	-	-	2738	2739	577	577	577	577
placement	253	235	1606	2513	561	669	5575	995
max	253	235	2738	2739	557	669	5575	995
CPU-512								
preproc.	-	-	3305	3602	122	122	122	122
placement	250	248	1491	2677	217	557	681	447
max	250	248	3305	3602	217	557	681	447

Table 3: Maximum memory usage of all programs on *CPU-628* and *CPU-512* data sets as reported by the *eval_resources* workflow of PEWO. Shown is *PSS* in MB for preprocessing and placement steps.

3.5 Run Time Large Showcase

Besides the results shown in the main manuscript, we performed run time evaluations for a large amount of query reads on the *tara-3748* data set. Here, we simulated $3748 \cdot 10^n$ reads for $n \in \{1, 10, 100, 1000, 10000\}$ for each of the 3748 reference sequences of the data set resulting in up to 37,480,000 query reads. We used weights of 12 and 16 and always used 30 threads. All experiments were carried out twice. We measured the placement time needed by *App-SpaM* with the `time` command on Linux. We report the results for `real` and `user`. `Real` is the wall clock time, thus the real time that elapsed during the execution of the program. This not only includes the time of the process, but also potentially time spent by the system or other processes. `User` is the CPU time spent on *App-SpaM* summed across all 30 cores.

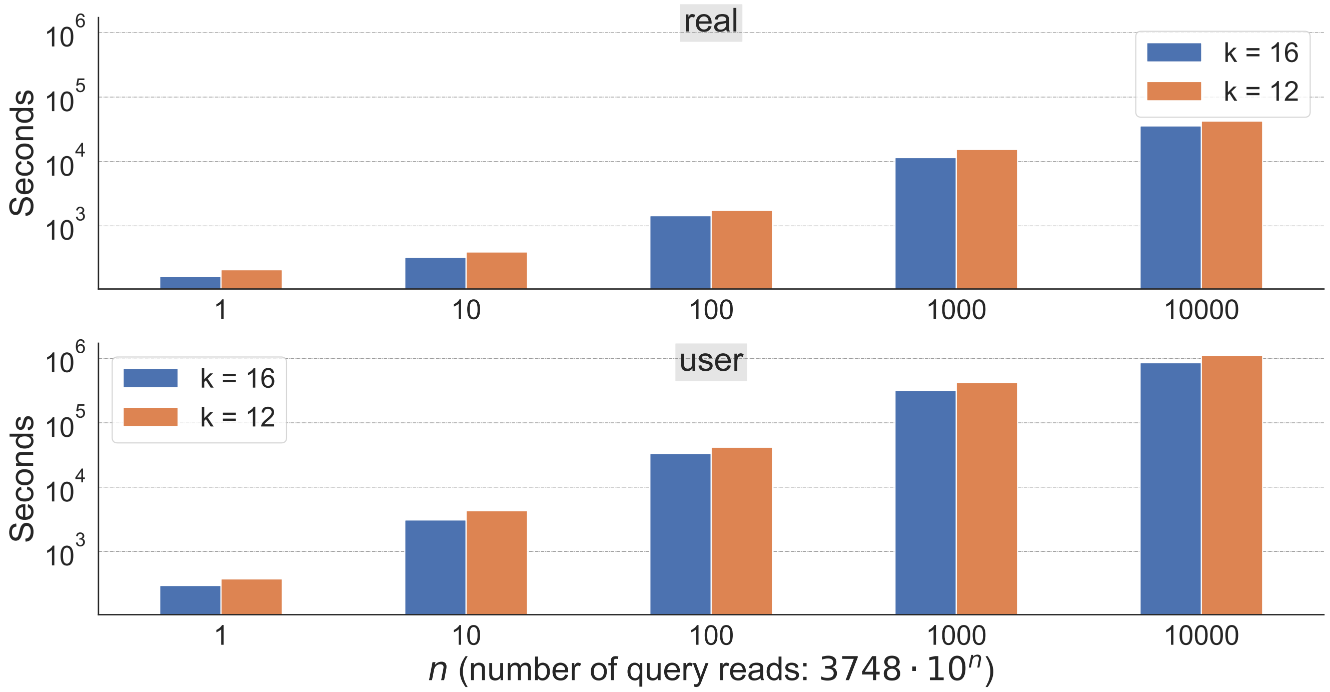

Figure 24: Run time of large test run on the *tara-3748* data set with increasingly many query reads. The number of query reads was varied between $3748 \cdot 10^n$, $n \in \{1, 10, 100, 1000, 10000\}$. Average of two runs is shown for each bar plot. Specific values given in table below.

Weight = 12

real	user	weight	threads	r	block
209	378	12	30	1	2000
208	375	12	30	1	2000
387	4398	12	30	10	2000
400	4240	12	30	10	2000
1693	41161	12	30	100	2000
1749	41957	12	30	100	2000
14649	400296	12	30	1000	2000
16083	440223	12	30	1000	2000
43607	1110812	12	30	10000	2000
41118	1097684	12	30	10000	2000

Weight = 16

real	user	weight	threads	r	block
160	301	16	30	1	2000
166	294	16	30	1	2000
343	3097	16	30	10	2000
304	3097	16	30	10	2000
1442	33963	16	30	100	2000
1437	32883	16	30	100	2000
11654	324631	16	30	1000	2000
11323	312966	16	30	1000	2000
35181	843120	16	30	10000	2000
36089	865720	16	30	10000	2000

4 Additional Results – ART

We extended the *PEWO PAC* workflow by using the program *ART* [5] that simulates sequencing reads for common sequencing platforms. In the *PAC* workflow, *PEWO* generates query reads by splitting the pruned reference sequences into non-overlapping segments of the specified query length and removes all resulting queries that are shorter than 50 *bp*. Instead, we performed additional accuracy evaluations with Illumina-simulated query reads by *ART*. *ART* uses a realistic model to simulate sequencing errors for the query reads. For these test runs we used again the three data sets *bac-150*, *hiv-104*, and *neotrop-512* with 50 prunings each. In every pruning run, and for every removed reference sequence, we used *ART* to simulate 50 query reads with length 150 *bp* with the Illumina *HiSeq 2500* error profile with default parameters. We could not find the explicit error profiles used in this case, but we expect that they were estimated similarly to other profiles given in the paper. Thus, we expect the reads to have roughly 0.0011 nucleotide substitutions per sequence position on average. The default for insertions is 0.00009 and for deletions is 0.00011.

The results that we obtained for query reads simulated with *ART* are shown in Fig. 25. Note that, unlike the default version of *PEWO*, the program *ART* simulates sequencing errors. As can be seen in the figure, the performance of *App-SpaM* with the placement heuristic *SpaM-4* is almost not affected by the introduced sequencing errors. All other programs show a significant drop in their placement accuracy if *ART* is used to simulate reads, compared to the simulated reads used in *PEWO* by default.

We are unsure about the reason for these pronounced results. In general, we think that *App-SpaM* is only little influenced by substitution errors by design of the spaced words. This is backed by the figure that shows only a small accuracy decrease from PEWO-reads to ART-reads. However, it poses the question why all other programs show such a large drop of accuracy. A possible explanation for the different test results on simulated reads with and without sequencing errors is as follows: Our *spaced word* approach is generally less affected by nucleotide mismatches than methods that rely on exact word matches or on exact alignments. Also, PEWO uses the program *hmmalign* to align queries to the reference MSA; in presence of simulated errors the hmm-profile-based alignments might lead to imprecise query alignments and hence inaccurate placements results. We are hoping for *PEWO* to support the simulation of sequencing errors in the query reads with *ART* or a similar program by default in the future. More thorough tests are needed to verify or refute the here presented results.

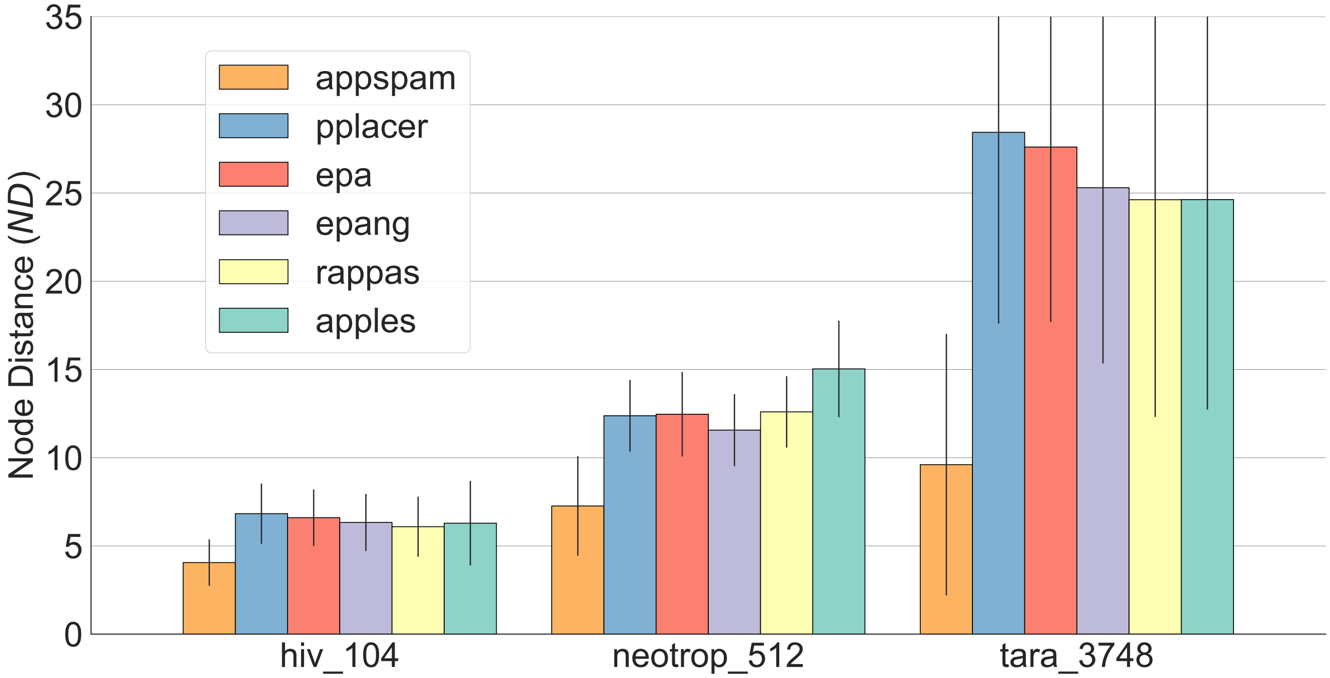

Figure 25: Average node distance for query reads of length 150 *bp* generated with *ART* on three data sets over $n = 50$ pruning events. Standard deviation across prunings are shown (black lines). Same parameters were used as in Subsec. 1.2.

References

[1] Metin Balaban, Shahab Sarmashghi, and Siavash Mirarab. APPLES: Fast distance-based phylogenetic placement. *Systematic Biology*, 69:566–578, 2020.

[2] Pierre Barbera, Alexey M. Kozlov, Lucas Czech, Benoit Morel, Diego Darriba, Tomás Flouri, and Alexandros Stamatakis. EPA-ng: Massively parallel evolutionary placement of genetic sequences. *Systematic Biology*, 68:365–369, 2019.

[3] Simon A. Berger, Denis Krompass, and Alexandros Stamatakis. Performance, accuracy, and web server for evolutionary placement of short sequence reads under maximum likelihood. *Systematic Biology*, 60:291–302, 2011.

[4] Lars Hahn, Chris-André Leimeister, Rachid Ounit, Stefano Lonardi, and Burkhard Morgenstern. *rasbhari*: optimizing spaced seeds for database searching, read mapping and alignment-free sequence comparison. *PLOS Computational Biology*, 12(10):e1005107, 2016.

[5] Weichun Huang, Leping Li, Jason R Myers, and Gabor T Marth. ART: a next-generation sequencing read simulator. *Bioinformatics*, 28:593–594, 2011.

[6] Anna Katharina Lau, Svenja Dörrer, Chris-André Leimeister, Christoph Bleidorn, and Burkhard Morgenstern. *Read-SpaM*: assembly-free and alignment-free comparison of bacterial genomes with low sequencing coverage. *BMC Bioinformatics*, 20:638, 2019.

[7] Chris-Andre Leimeister, Jendrik Schellhorn, Svenja Dörrer, Michael Gerth, Christoph Bleidorn, and Burkhard Morgenstern. Prot-SpaM: Fast alignment-free phylogeny reconstruction based on whole-proteome sequences. *GigaScience*, 8:giy148, 2019.

[8] Chris-André Leimeister, Salma Sohrabi-Jahromi, and Burkhard Morgenstern. Fast and accurate phylogeny reconstruction using filtered spaced-word matches. *Bioinformatics*, 33:971–979, 2017.

[9] Benjamin Linard, Nikolai Romashchenko, Fabio Pardi, and Eric Rivals. PEWO: a collection of workflows to benchmark phylogenetic placement. *Bioinformatics*, btaa657, 2020.

[10] Benjamin Linard, Krister Swenson, and Fabio Pardi. Rapid alignment-free phylogenetic identification of metagenomic sequences. *Bioinformatics*, 35:3303–3312, 2019.

[11] Frédéric Mahé, Colomban De Vargas, David Bass, and et. al. Parasites dominate hyperdiverse soil protist communities in Neotropical rainforests. *Nature Ecology and Evolution*, 2017.

[12] Frederick A. Matsen, Robin B. Kodner, and E. Virginia Armbrust. pplacer: linear time maximum-likelihood and Bayesian phylogenetic placement of sequences onto a fixed reference tree. *BMC Bioinformatics*, 11:538, 2010.

[13] Burkhard Morgenstern. Sequence comparison without alignment: The SpaM approaches. In Kazutaka Katoh, editor, *Multiple Sequence Alignment*, Methods in Molecular Biology. Springer, 2020. to appear.

[14] Sujatha Srinivasan, Noah G. Hoffman, Martin T. Morgan, and et. al. Bacterial communities in women with bacterial vaginosis: High resolution phylogenetic analyses reveal relationships of microbiota to clinical criteria. *PLoS ONE*, 2012.

[15] Shinichi Sunagawa, Luis Pedro Coelho, Samuel Chaffron, and et. al. Structure and function of the global ocean microbiome. *Science*, 2015.
